# Iron–quercetin nanocomplex preconditioning reprograms the mesenchymal stem cell secretome to drive angiogenic, fibroblast and immunoregulatory wound repair

**DOI:** 10.1093/rb/rbag132

**Published:** 2026-06-15

**Authors:** Jiraporn Kantapan, Phattarawadee Innuan, Donraporn Daranarong, Sittiruk Roytrakul, Krit Jaikumkao, Gwenaël Rolin, Céline Viennet-Steiner, Worapong Khaodee, Padchanee Sangthong, Kittichai Wantanajittikul, Winita Punyodom, Nathupakorn Dechsupa

**Affiliations:** Molecular Imaging and Therapy Research Unit, Department of Radiologic Technology, Faculty of Associated Medical Sciences, Chiang Mai University, Chiang Mai 50200, Thailand; Department of Radiologic Technology, Faculty of Associated Medical Sciences, Chiang Mai University, Chiang Mai 50200, Thailand; Molecular Imaging and Therapy Research Unit, Department of Radiologic Technology, Faculty of Associated Medical Sciences, Chiang Mai University, Chiang Mai 50200, Thailand; Department of Radiologic Technology, Faculty of Associated Medical Sciences, Chiang Mai University, Chiang Mai 50200, Thailand; Multidisciplinary Research Institute, Chiang Mai University, Chiang Mai 50200, Thailand; Center of Excellence in Materials Science and Technology, Chiang Mai University, Chiang Mai 50200, Thailand; National Center for Genetic Engineering and Biotechnology, National Science and Technology Development Agency, Pathum Thani 12120, Thailand; Department of Radiologic Technology, Faculty of Associated Medical Sciences, Chiang Mai University, Chiang Mai 50200, Thailand; INSERM CIC-1431, CHU Besançon, Besançon F-25000, France; Université Marie et Louis Pasteur, UM RIGHT, Besançon F-25000, France; INSERM CIC-1431, CHU Besançon, Besançon F-25000, France; Faculty of Allied Health Sciences, Burapha University, Chon Buri 20131, Thailand; Department of Chemistry, Faculty of Science, Chiang Mai University, Chiang Mai 50200, Thailand; Department of Radiologic Technology, Faculty of Associated Medical Sciences, Chiang Mai University, Chiang Mai 50200, Thailand; Center of Excellence in Materials Science and Technology, Chiang Mai University, Chiang Mai 50200, Thailand; Department of Chemistry, Faculty of Science, Chiang Mai University, Chiang Mai 50200, Thailand; Molecular Imaging and Therapy Research Unit, Department of Radiologic Technology, Faculty of Associated Medical Sciences, Chiang Mai University, Chiang Mai 50200, Thailand; Department of Radiologic Technology, Faculty of Associated Medical Sciences, Chiang Mai University, Chiang Mai 50200, Thailand

**Keywords:** iron–quercetin nanocomplex, secretome, wound healing, angiogenesis, immunomodulation

## Abstract

Chronic wounds remain a major clinical challenge characterized by impaired angiogenesis, persistent inflammation and dysfunctional stromal responses. Increasing evidence indicates that the therapeutic effects of mesenchymal stem cells (MSCs) are largely mediated by paracrine signaling, making MSC-derived secretomes promising cell-free regenerative therapies. However, conventional culture conditions do not fully harness MSC plasticity to enhance secretion of pro-regenerative factors. In this study, we investigated an iron–quercetin nanocomplex (IronQ) as a biocompatible preconditioning strategy to improve the therapeutic secretome of adipose-derived MSCs (ADSCs). IronQ priming preserved cell viability and phenotypic integrity while allowing efficient intracellular uptake. Transcriptomic analysis demonstrated coordinated ADSC reprogramming, with enrichment of pathways related to extracellular matrix organization, angiogenesis, immune regulation, iron homeostasis and tissue morphogenesis. Consistently, IronQ-preconditioned ADSC secretomes showed increased levels of key trophic and immunomodulatory factors, including VEGF-A, HGF, EGF, FGF-2, PDGF-AA, SDF-1α, G-CSF, CCL-2 and IL-10. Functionally, the conditioned secretome enhanced endothelial proliferation and tube formation, promoted fibroblast migration and activation, and induced macrophage polarization toward a reparative M2 phenotype *in vitro*. Collectively, these findings identify IronQ as an effective secretome-modulating strategy with strong translational potential for scalable, cell-free treatment of chronic and diabetic wounds.

## Graphical Abstract

**Graphical Abstract:**
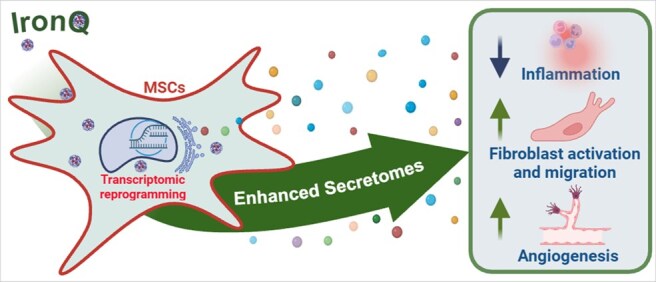

## Introduction

Chronic non-healing wounds, particularly those associated with diabetes, represent a persistent clinical challenge with profound health and socioeconomic consequences worldwide [1]. Despite advances in wound care, many patients experience delayed healing driven by impaired angiogenesis, persistent inflammation and dysfunctional stromal cell responses. In this context, regenerative therapies have attracted increasing attention as a means to restore the complex cellular crosstalk required for effective tissue repair. Increasing evidence suggests that the regenerative effects of cell-based therapies arise largely from the release of paracrine bioactive factors rather than long-term cellular engraftment or direct differentiation [2, 3]. These paracrine cues orchestrate key processes essential for wound healing, including neovascularization, immune resolution and coordinated migration and proliferation of keratinocytes and fibroblasts.

Mesenchymal stem cells (MSCs) have been widely investigated for their regenerative potential in acute and chronic wounds, diabetic ulcers, radiation injuries and other tissue repair settings, with encouraging outcomes in both preclinical and clinical studies [4–6]. Importantly, accumulating evidence demonstrates that the conditioned medium (CM) or supernatant derived from MSC cultures can recapitulate many of these therapeutic effects, underscoring the central role of the MSC secretome in mediating tissue regeneration [7]. This realization has catalysed a paradigm shift from cell replacement toward secretome-based therapies. Composed of cytokines, chemokines, growth factors, extracellular vesicles and matrix-associated proteins, the MSC secretome orchestrates tissue repair through complex and dynamic intercellular signaling networks [8, 9]. Delivery of CM, as an acellular therapeutic modality, retains the key regenerative functions of MSCs while circumventing major limitations of live-cell therapies, including immune incompatibility, tumorigenic risk, vascular embolization and complex handling requirements [10, 11]. Moreover, CM can be sterilized, standardized, lyophilized and batch-controlled, enabling scalable manufacturing and reproducible quality features that strongly favor clinical translation [12–14]. These attributes position secretome-based therapies as practical, off-the-shelf bioactive materials for regenerative wound care.

Among MSC sources, adipose-derived MSCs (ADSCs) are particularly attractive for secretome-based applications due to their abundance, ease of harvest and robust paracrine activity [15, 16]. Compared with bone marrow-derived MSCs (BM-MSCs), ADSCs can be obtained through less invasive procedures and generally provide higher cell yield and proliferative capacity. In addition, ADSCs exhibit strong paracrine and angiogenic activity. Their secretomes are enriched in growth factors and immunomodulatory mediators relevant to wound healing and tissue regeneration, including vascular endothelial growth factor (VEGF), hepatocyte growth factor (HGF), fibroblast growth factors (FGFs), insulin-like growth factor (IGF) and anti-inflammatory cytokines. Collectively, these factors promote angiogenesis, fibroblast activation, extracellular matrix (ECM) remodeling and re-epithelialization [17–19]. These characteristics make ADSCs particularly suitable for clinically translatable secretome-based therapeutic strategies targeting chronic and diabetic wounds. However, under conventional culture conditions, MSCs do not fully exploit their intrinsic plasticity, resulting in secretomes that may be suboptimal for challenging regenerative environments such as diabetic wounds. To address this limitation, a range of preconditioning strategies including hypoxia, inflammatory cytokines, pharmacological agents and nanomaterials have been developed to ‘tune’ MSC secretory output toward enhanced therapeutic efficacy [20, 21]. Among these, nanomaterial-based priming has emerged as a particularly promising approach due to its tunability, scalability and ability to modulate intracellular signaling pathways without permanent genetic modification.

In the present study, we investigated the iron–quercetin nanocomplex (IronQ) as a biologically active iron–polyphenol nanoplatform with functions extending beyond traditional diagnostic applications. Previous studies have demonstrated that IronQ exhibits efficient cellular internalization and favorable biocompatibility while also serving as a T1-weighted magnetic resonance imaging (MRI) contrast agent across multiple cell types [22–24]. Importantly, emerging evidence indicates that IronQ can modulate intracellular redox balance and signaling pathways implicated in angiogenesis, inflammation, and tissue repair. In earlier work, we showed that secretomes derived from IronQ-primed peripheral blood mononuclear cells (PBMCs) significantly enhanced regenerative responses in diabetic wound-healing models, supporting the feasibility of IronQ as an accessible priming platform for cell-free regenerative therapies [25]. Furthermore, IronQ-pretreated MSCs have shown superior therapeutic efficacy in diverse disease models, including enhanced neuroprotection in intracerebral hemorrhage [26] and improved recovery from cisplatin-induced acute kidney injury via activation of the HGF/c-Met axis [27]. Collectively, these findings suggest that IronQ functions not merely as a passive iron carrier or imaging probe, but as a bioactive modulator capable of reprogramming MSC function. Despite these promising observations, critical gaps remain. Whether IronQ-mediated enhancement of MSC therapeutic function is conserved across stromal-driven regenerative processes such as wound healing, and how IronQ reshapes MSC biology at the transcriptomic and secretomic levels, remain poorly defined. In particular, a systematic mechanistic understanding of how IronQ preconditioning translates intracellular modulation into a functionally coherent, pro-regenerative secretome is lacking.

Taken together, these observations led us to hypothesize that IronQ preconditioning can induce a therapeutically enhanced secretome from ADSCs, characterized by enriched trophic, pro-angiogenic and immunomodulatory factors relevant to wound healing. In the present study, we systematically investigated the effects of IronQ on the MSC transcriptome to elucidate signaling pathways involved in IronQ-treated ADSCs, characterized changes in secretory profiles and evaluated the functional consequences of IronQ-induced MSC secretomes using *in vitro* surrogates of tissue repair, including endothelial tube formation, fibroblast migration and cytokine profiling. By focusing on a cell-free, IronQ-tuned MSC secretome, this work aims to advance a scalable and translational strategy for regenerative therapy, with particular relevance to chronic and diabetic wound healing.

## Materials and methods

### Preparation of the IronQ

The IronQ was synthesized and comprehensively characterized in our previous work [22, 24]. The same formulation, synthesis procedure and purification protocol were used in the present study without modification. As previously reported, IronQ consists of nanoscale iron–polyphenol complexes exhibiting favorable colloidal stability and biocompatibility under aqueous and physiological conditions. To confirm the physicochemical properties of the IronQ formulation used in the present study, UV–Vis spectroscopy, dynamic light scattering (DLS), zeta potential analysis, scanning electron microscopy (SEM) and energy-dispersive X-ray (EDX) spectroscopy were performed. The UV–Vis spectrum demonstrated characteristic absorption peaks at 294 nm and within the 500–700 nm range, consistent with the formation of iron(III)–quercetin complexes. The hydrodynamic diameter of IronQ was determined to be 225.90 ± 21.43 nm, while the zeta potential was measured at −18.20 ± 0.66 mV, indicating moderate colloidal stability in aqueous suspension (Supplementary Figure S1A–C). SEM analysis revealed nanoscale particles with irregular amorphous morphology (Supplementary Figure S1D). In addition, EDX elemental analysis confirmed the presence of major constituent elements including carbon (C), oxygen (O), sodium (Na), chlorine (Cl) and iron (Fe), supporting successful formation of the IronQ (Supplementary Figure S1E).

Prior to use, lyophilized IronQ was reconstituted in distilled water to obtain a 2 mg mL^−1^ stock solution. The preparation was subsequently passed through a 0.2 µm sterile syringe filter and maintained at 4°C before experimental application. Because the synthesis, physicochemical properties and *in vivo* biocompatibility of IronQ have been rigorously established in prior peer-reviewed studies, the present work focuses on its biological priming effects on ADSCs and secretome-mediated regenerative functions rather than reiterating material characterization.

### ADSCs culture and immunophenotyping

Human ADSCs (PCS-500-011^™^) were purchased from the American Type Culture Collection (ATCC, Manassas, VA, USA). Cells were maintained in low-glucose Dulbecco’s Modified Eagle Medium (DMEM; Caisson Laboratories, Smithfield, UT, USA) supplemented with 10% fetal bovine serum (FBS; Gibco, Waltham, MA, USA), 1% L-glutamine and 1% penicillin–streptomycin. Cultures were incubated at 37°C under humidified 5% CO_2_ conditions and subcultured when reaching approximately 70–80% confluence.

For immunophenotypic analysis, ADSCs were collected and resuspended in phosphate-buffered saline (PBS) supplemented with 0.1% bovine serum albumin (BSA) to a final density of 5 × 10^5^ cells per sample. Aliquots (100 µL) of the cell suspension were incubated with fluorochrome-conjugated monoclonal antibodies against MSC surface markers, including FITC-conjugated anti-CD34 (Elabscience), Alexa Fluor 488-conjugated anti-CD73 (eBioscience), PE/Cy5.5-conjugated anti-CD90 (eBioscience), PE-conjugated anti-CD105 (eBioscience) and PE-conjugated anti-CD45 (eBioscience). Corresponding isotype-matched IgG controls were included for each fluorescence channel. Antibody staining was performed at 4°C for 30 min in the dark. After incubation, cells were rinsed twice with PBS supplemented with 0.1% BSA and prepared for flow cytometric evaluation. Samples were acquired using a Beckman Coulter Epics XL-MCL flow cytometer (Beckman Coulter, CA, USA), and the resulting data were processed with CytExpert^™^ software (version 2.5, Beckman Coulter). Cell populations were gated based on forward- and side-scatter characteristics to exclude debris and cell aggregates.

### Cell lines and cell cultures

Human dermal fibroblasts (HDFs) and human umbilical vein endothelial cells (HUVECs) were purchased from ATCC (Manassas, VA, USA). HDFs were maintained in DMEM (Gibco, Waltham, MA, USA) supplemented with 10% FBS, 2 mM L-glutamine and 1% penicillin–streptomycin. HUVEC cultures were maintained in endothelial growth medium (EGM) prepared using DMEM/F-12 supplemented with 10% FBS, 1% penicillin–streptomycin and endothelial growth supplements including epidermal growth factor (EGF, 5 ng mL^−1^), basic FGF (bFGF, 10 ng mL^−1^), IGF-1 (20 ng mL^−1^), VEGF (0.5 ng mL^−1^), heparin (22.5 µg mL^−1^) and hydrocortisone (0.2 µg mL^−1^).

The human monocytic leukemia cell line THP-1 (ATCC® TIB-202^™^) was cultured in RPMI-1640 medium containing 10% FBS, 2 mM L-glutamine and 1% penicillin–streptomycin. To induce macrophage-like differentiation, THP-1 cells were exposed to phorbol 12-myristate 13-acetate (PMA; 50 ng mL^−1^) for 48 h. After PMA stimulation, cells were rinsed three times with PBS and subsequently maintained in PMA-free complete RPMI-1640 medium for an additional 24 h before downstream experiments. All cultures were maintained at 37°C in a humidified atmosphere containing 5% CO_2_.

### Cytotoxicity assay

The cytocompatibility of the IronQ toward ADSCs was examined using both MTT metabolic activity and lactate dehydrogenase (LDH) release assays. ADSCs were seeded into 96-well plates at 5 × 10^3^ cells per well in complete DMEM supplemented with 10% FBS and 1% penicillin–streptomycin. Following 24 h of incubation, the medium was replaced with fresh culture medium containing various concentrations of IronQ (0–1000 µg mL^−1^). Cells were then further incubated for 24 h prior to analysis. For metabolic viability assessment, cells were rinsed twice with PBS before incubation with 100 µL of DMEM containing MTT reagent (0.5 mg mL^−1^; Sigma-Aldrich, St. Louis, MO, USA). After 4 h at 37°C, the resulting formazan crystals were dissolved in dimethyl sulfoxide (DMSO) following removal of the supernatant. Absorbance was recorded at 560 nm using a BioTek^™^ Cytation 5 microplate reader (Agilent Technologies, Santa Clara, CA, USA). Cell viability was normalized to untreated controls and expressed as percentage viability.

Membrane damage was evaluated by measuring LDH release into the culture medium using a commercial LDH cytotoxicity assay kit (Merck, Darmstadt, Germany) according to the manufacturer’s protocol. Briefly, culture supernatants (50–100 µL) collected after IronQ exposure were transferred to a separate 96-well plate and mixed with the reaction reagent. Samples were incubated for 20–30 min at room temperature in the absence of light before absorbance measurement at 490 nm with a reference wavelength of 620 nm using the same microplate reader. Maximum LDH release was determined using kit-provided lysis buffer-treated wells, whereas spontaneous release was obtained from untreated controls. Cytotoxicity was calculated according to the manufacturer’s equation and expressed as percentage LDH release.

In parallel, potential alterations in ADSC morphology and mesenchymal immunophenotype following IronQ exposure were evaluated using bright-field microscopy and flow cytometric analysis of canonical MSC surface markers, as described in Section ‘ADSCs culture and immunophenotyping’.

### IronQ labeling of ADSCs and assessment of labeling efficiency by Prussian blue staining and inductively coupled plasma–optical emission spectrometry

ADSCs were seeded into 6-well plates at a density of 1 × 10^6^ cells per well in complete DMEM containing 10% FBS and 1% penicillin–streptomycin. After allowing sufficient time for attachment, cells were exposed to IronQ at concentrations of 0, 125, 250, 500, 750 or 1000 µg mL^−1^ for 24 h under standard culture conditions (37°C, 5% CO_2_). Following treatment, culture medium was discarded and cells were washed three times with PBS to eliminate residual extracellular IronQ. Intracellular iron accumulation was assessed qualitatively using Prussian blue staining and quantitatively by inductively coupled plasma–optical emission spectrometry (ICP-OES). For histochemical staining, cells were fixed in 4% paraformaldehyde for 20 min at room temperature and subsequently incubated with freshly prepared Prussian blue working solution (Perls’ reagent; G1422, Solarbio, China) for 30 min. After thorough washing with distilled water, intracellular ferric iron deposits were visualized as blue cytoplasmic staining using an inverted microscope (ECLIPSE Ti, Nikon, Japan) equipped with a DS-Ri2 camera. Representative micrographs were captured at 100× total magnification (10× objective lens) using NIS-Elements software (version F4.30.01). For quantitative iron determination, treated ADSCs were collected and subjected to acid digestion in 70% nitric acid at 60°C for 4 h. Digested samples were subsequently diluted with deionized water to obtain a final nitric acid concentration of 2% prior to ICP-OES analysis. Intracellular iron levels were normalized to total cell number and expressed as iron content per cell.

### IronQ preconditioning of ADSCs and preparation of ADSC-derived secretomes

ADSCs were expanded in T75 culture flasks under standard growth conditions until approximately 70–80% confluence was achieved. To minimize variability in CM production, cells were seeded at equivalent densities and maintained under identical culture conditions prior to treatment. ADSCs were subsequently exposed to either IronQ (250 µg mL^−1^) or vehicle control for 24 h. Following treatment, cultures were washed three times with PBS to remove residual IronQ and serum-derived components. Cells were then incubated in serum-free DMEM for an additional 24 h to allow secretome collection. At the time of CM harvest, cell viability and total cell number were evaluated using trypan blue exclusion analysis to confirm comparable cell densities between control and IronQ-treated groups. Collected conditioned media were centrifuged at 2000 rpm for 30 min to eliminate suspended cells and cellular debris, followed by filtration through a sterile 0.22 µm syringe filter for further clarification. CM obtained from IronQ-treated ADSCs was designated as IronQ-CM, whereas medium collected from untreated cells was referred to as control CM (C-CM). Prepared conditioned media were either applied immediately in downstream assays or stored at −80°C until use. For all functional experiments, conditioned media were administered using equivalent volume ratios under standardized experimental conditions.

### RNA-sequencing analysis and bioinformatic analysis

Total RNA was isolated from untreated ADSCs and IronQ-treated ADSCs (24 h exposure) using the NucleoSpin® RNA Kit (Macherey-Nagel, Düren, Germany) according to the manufacturer’s instructions. Residual genomic DNA contamination was removed by on-column digestion with RNase-free rDNase. RNA quantity and purity were evaluated using a NanoDrop spectrophotometer (BioDrop^™^ µLITE, Cambridge, UK), while RNA integrity was assessed based on A260/A280 absorbance ratios.

For transcriptomic analysis, mRNA was enriched from total RNA using oligo(dT)-conjugated magnetic beads and fragmented prior to complementary DNA (cDNA) synthesis. First-strand cDNA was generated using random hexamer primers, followed by second-strand synthesis to obtain double-stranded cDNA. Sequencing libraries were prepared through end repair, A-tailing, adaptor ligation, PCR amplification and purification steps. Libraries were quantified, pooled at equal molar ratios and subjected to paired-end sequencing (150 bp) using an Illumina sequencing platform. Raw sequencing reads underwent quality assessment using FastQC. Adapter sequences, poly-N-containing reads and low-quality reads were removed prior to downstream analysis. Filtered clean reads were evaluated according to Q20, Q30 and GC-content metrics and subsequently aligned to the human reference genome (GRCh38) using HISAT2 (version 2.0.5). Gene-level read quantification was performed with featureCounts (version 1.5.0-p3), and transcript abundance was normalized as fragments per kilobase of transcript per million mapped reads (FPKM). Principal component analysis (PCA) was carried out to assess transcriptomic variation between experimental groups. Differential expression analysis was performed using the DESeq2 R package (version 1.20.0), which employs a negative binomial statistical model. Genes with an adjusted *P-*value (*Q*-value) < 0.05 and |log_2_ fold change| ≥ 1 were defined as significantly differentially expressed. Visualization of transcriptomic patterns, including volcano plots, heatmaps and Venn diagrams, was performed using the NovoMagic online analysis platform (Novogene).

Functional enrichment analysis of differentially expressed genes (DEGs) was conducted using Gene Ontology (GO) and Kyoto Encyclopedia of Genes and Genomes (KEGG) databases through the NovoMagic platform, with significance thresholds set at Q < 0.05. Protein–protein interaction (PPI) networks were generated using the STRING database and visualized in Cytoscape (version 3.8.2). Highly interconnected clusters identified within the PPI network were further analysed using the Metascape platform to identify enriched biological pathways and molecular functions.

### Characterization of cytokines and growth factors in ADSC secretome

Cytokine and growth factor profiles of ADSC secretomes were analysed using the Invitrogen^™^ Human ProcartaPlex^™^ Mix & Match Panel (Thermo Fisher Scientific, Cat. No. PPX-05-MX2XA49), based on xMAP® bead-based multiplex technology. The panel simultaneously quantified HGF, IGF, EGF, FGF2, G-CSF, M-CSF, VEGF-A, TGF-β, PDGF-AA, PDGF-BB, SDF-1, CCL-2 (MCP-1), IL-6 and IL-10. Conditioned media were thawed on ice, clarified by centrifugation at 10 000*g* for 10 min and kept on ice until analysis. All reagents were prepared according to the manufacturer’s instructions. Wash buffer (10×) was diluted to 1× using deionized water. Lyophilized standard mixtures were reconstituted in the same culture medium used for sample collection, vortexed briefly, centrifuged and equilibrated on ice. A 7-point, 1:4 serial dilution was prepared to generate standard curves for each analyte, along with a background control. Magnetic bead mixes were vortexed and 50 μL was added to each assay well. Standards, background and samples were loaded in duplicate (50 μL/well). Plates were incubated on a shaker (800 rpm for 1 min, then 600 rpm for 2 h) at room temperature, protected from light. Wash steps were performed using a magnetic plate separator. Subsequently, 25 μL of detection antibody was added and incubated for 30 min, followed by washing and incubation with 50 μL streptavidin–phycoerythrin (PE) for 30 min. After final washes, 120 μL of reading buffer was added and plates were shaken at 800 rpm for 5 min. Plates were analysed using a Luminex 200 (LX200) system with xPONENT® 3.1 software, following manufacturer-recommended calibration and verification procedures. Analyte concentrations were calculated from standard curves and expressed as pg/mL.

### Characterization of soluble protein in ADSC secretome by mass spectrometry

Soluble proteins present in ADSC-derived secretomes were profiled using liquid chromatography–tandem mass spectrometry (LC–MS/MS). Protein concentrations were first determined by the Lowry assay using BSA as a calibration standard [28]. Equivalent amounts of protein from each sample were subjected to in-solution digestion prior to mass spectrometric analysis. Protein samples were resuspended in 10 mM ammonium bicarbonate (AMBIC), reduced with 5 mM dithiothreitol (DTT) at 60°C for 1 h and subsequently alkylated with 15 mM iodoacetamide (IAA) prepared in 10 mM AMBIC for 45 min at room temperature in the dark. Proteins were digested overnight at 37°C using sequencing-grade porcine trypsin (Promega) at an enzyme-to-protein ratio of 1:20. Generated peptides were desalted, concentrated, and reconstituted in 0.1% formic acid before LC–MS/MS analysis.

Peptide separation and mass analysis were performed using a ZenoTOF 7600 mass spectrometer (SCIEX, Framingham, MA, USA) coupled to an Ultimate 3000 nano/capillary LC system (Thermo Fisher Scientific). To ensure analytical reproducibility, each biological sample was analysed in technical triplicate (*n* = 9 spectra per experimental condition). Raw mass spectrometry data were processed using MaxQuant software (version 2.5.0.0) [29], and peptide identification was carried out with the integrated Andromeda search engine against the Homo sapiens UniProt reference proteome (downloaded 14 January 2025).

Label-free quantification (LFQ) was performed using standard MaxQuant settings, including trypsin specificity with a maximum of two missed cleavages. Carbamidomethylation of cysteine was specified as a fixed modification, whereas methionine oxidation and protein N-terminal acetylation were considered variable modifications. The main peptide mass tolerance was set to 6 ppm. Protein identification required peptides of at least seven amino acids in length and a minimum of two peptides per protein, including one unique peptide. False discovery rates (FDRs) for peptide and protein identification were controlled at 1% using a reversed decoy database approach. LFQ intensity values were log_2_-transformed and quantile-normalized before statistical analysis to improve comparability across samples and reduce heteroscedasticity. Technical replicates containing excessive missing values were excluded according to predefined quality criteria, and only proteins detected in at least two out of three replicates were retained for downstream analysis. Missing values were imputed using the mean intensity of the corresponding observed replicates.

Differential protein abundance between groups was evaluated using Welch’s two-sample *t*-test. For each protein, mean intensity, SD, log_2_ fold change (log_2_FC), raw *P-*value and Benjamini–Hochberg-adjusted *P-*value were calculated. Proteins with raw *P* < 0.05 were considered significant for exploratory analyses. Data visualization, including heatmaps, volcano plots and PCA, was performed using MetaboAnalyst version 6.0 [30]. PPI networks were generated using STRING database version 12.0 [31], followed by GO and KEGG enrichment analyses. Statistical analyses and graphical visualization were performed in R (version 4.5.1) using CRAN and Bioconductor packages, including pheatmap, EnhancedVolcano and ggplot2.

### Cell proliferation assay

HDFs and HUVECs were seeded into 96-well plates at predetermined optimal densities and maintained overnight to allow cell attachment under standard culture conditions. Each experimental condition was performed using six technical replicates. After attachment, cells were exposed for 24, 72 or 120 h to culture media containing 50% (v/v) CM derived from ADSC secretomes, including CM from untreated ADSCs (C-CM) and IronQ-preconditioned ADSCs (IronQ-CM). Serum-free medium served as the negative control, whereas DMEM supplemented with 10% FBS was used as the positive control. Cell proliferation was evaluated at each time point using the Cell Counting Kit-8 (CCK-8; Abbkine, Wuhan, China). Briefly, 10 µL of CCK-8 reagent was added to each well containing 100 µL of fresh culture medium supplemented with 10% FBS, including blank control wells. Plates were then incubated for 2 h at 37°C in the absence of light before absorbance measurement at 450 nm using a BioTek^™^ Cytation 5 microplate reader (Agilent Technologies, Santa Clara, CA, USA). Relative optical density (OD) values were calculated and normalized to the corresponding control groups to determine the effects of ADSC-derived conditioned media on HDF and HUVEC proliferation.

### Cell migration by a scratch wound healing assay

The migratory effects of ADSC-derived secretomes were evaluated using a scratch wound healing assay. Cells were cultured until a continuous confluent monolayer was formed prior to wound generation. A straight scratch was created across the cell monolayer using a sterile pipette tip to generate a reproducible cell-free region. Detached cells and residual debris were carefully removed by washing with PBS. Following scratch formation, cultures were maintained in serum-free medium containing 50% (v/v) CM derived from untreated ADSCs (C-CM) or IronQ-preconditioned ADSCs (IronQ-CM). Serum-free medium alone was used as the negative control, whereas DMEM supplemented with 10% FBS served as the positive control. Cells were incubated at 37°C in a humidified 5% CO_2_ atmosphere throughout the experiment. Images of the wounded region were acquired immediately after scratch generation (0 h) and again after 24 h using an inverted phase-contrast microscope (ECLIPSE Ts2, Nikon, Tokyo, Japan). Migration activity was quantified by measuring wound closure over time using ImageJ software (version 1.52v; National Institutes of Health, Bethesda, MD, USA). The percentage of wound closure relative to the initial scratch area was calculated and used as an indicator of cellular migratory capacity under each experimental condition.

### Cell migration assay using Transwell chambers

To further investigate directed cell migration in response to ADSC-derived secretomes, Transwell migration assays were conducted using Millicell® 24-well inserts equipped with 8-µm pore polycarbonate membranes (Merck Millipore, USA). Prior to the assay, cells were maintained in serum-free medium for 24 h to reduce baseline migratory activity. Cells were subsequently collected, resuspended in serum-free medium and seeded into the upper compartments of the Transwell inserts at appropriate densities. The lower chambers contained 700 µL of culture medium supplemented with 50% (v/v) CM derived from untreated ADSCs (C-CM) or IronQ-preconditioned ADSCs (IronQ-CM), which served as chemoattractant stimuli. Cultures were incubated for 24 h at 37°C under humidified 5% CO_2_ conditions to permit migration through the porous membrane. Following incubation, cells remaining on the upper membrane surface were carefully removed with cotton swabs. Migrated cells attached to the lower membrane surface were fixed with 4% paraformaldehyde in PBS and stained using crystal violet for 15 min at room temperature. Stained cells were visualized using an inverted microscope (ECLIPSE Ts2, Nikon, Tokyo, Japan), and migrated cells were quantified by counting multiple randomly selected microscopic fields per insert. All experiments were performed in triplicate, and migration activity was expressed as the relative number of migrated cells per field.

### *In vitro* tube formation assay

The pro-angiogenic effects of ADSC-derived secretomes were investigated using an *in vitro* endothelial tube formation assay performed on Matrigel matrix (Thermo Fisher Scientific, Waltham, MA, USA). Matrigel was thawed under cold conditions, dispensed into 96-well plates and incubated at 37°C to allow gel polymerization prior to cell seeding. HUVECs were seeded onto Matrigel-coated wells at a density of 5 × 10^4^ cells per well and cultured under the following conditions: (i) low-serum DMEM (basal medium), (ii) 50% EGM (control), (iii) 100% EGM (positive control), (iv) 50% control ADSCs-CM mixed with 50% EGM (C-CM) and (v) 50% IronQ-preconditioned ADSCs-CM mixed with 50% EGM (IronQ-CM). For the CM groups, a 1:1 (v/v) mixture of CM and EGM was used to maintain endothelial cell viability while enabling assessment of the relative pro-angiogenic effect of the secretome under standardized growth factor conditions. Cultures were maintained at 37°C under humidified 5% CO_2_ conditions, and endothelial network formation was evaluated at 4, 8 and 12 h using an inverted microscope (ECLIPSE Ts2, Nikon, Tokyo, Japan). Representative micrographs were obtained from six randomly chosen fields per well at each observation time point. Quantitative assessment of angiogenic network formation was carried out using WimTube image analysis software (Wimasis GmbH, Munich, Germany), including measurements of total tube length, junction number, loop formation and branching structures. To account for the basal angiogenic activity induced by growth factor supplementation, all quantitative data were normalized to the EGM control condition (50% EGM), allowing assessment of relative angiogenic enhancement beyond the baseline. For each condition, measurements were obtained from triplicate wells across a minimum of three independent experiments. Quantitative results are reported as mean values with corresponding standard deviations.

### Effect of ADSC-derived secretomes on M2 macrophage polarization

To assess the immunomodulatory effects of ADSC-derived secretomes on macrophage polarization, M0 macrophages were generated from THP-1 cells as described in Section ‘Cell lines and cell cultures’ and subsequently exposed to conditioned media from ADSCs. Macrophages were treated with CM derived from untreated ADSCs (C-CM) or IronQ-preconditioned ADSCs (IronQ-CM). Conditioned media were mixed with fresh complete culture medium at a 1:1 (v/v) ratio and applied to M0 macrophages for 48 h to induce phenotypic polarization. Following incubation, macrophages were harvested for phenotypic and molecular analyses. Cell surface expression of macrophage polarization markers was evaluated by flow cytometry, while total RNA was extracted for quantitative real-time PCR (qRT-PCR) analysis of M1- and M2-associated genes. In parallel, culture supernatants were collected and stored at −80°C for subsequent cytokine quantification by enzyme-linked immunosorbent assay (ELISA).

### Quantitative real-time polymerase chain reaction

Total RNA was isolated using the NucleoSpin® RNA Kit (Macherey–Nagel, Düren, Germany) according to the manufacturer’s recommendations. Genomic DNA contamination was removed during the extraction procedure through on-column DNase treatment. Purified RNA samples were subsequently reverse transcribed into complementary DNA (cDNA) using ReverTra Ace^™^ qPCR RT Master Mix with gDNA Remover (TOYOBO, Osaka, Japan). qRT-PCR analyses were carried out using a CFX Connect^™^ Real-Time PCR Detection System (Bio-Rad, Hercules, CA, USA) with THUNDERBIRD^™^ Next SYBR® qPCR Mix (TOYOBO, Osaka, Japan). Amplification reactions were prepared using cDNA as the template. Thermal cycling conditions included an initial denaturation step at 95°C for 60 s, followed by 40 amplification cycles consisting of denaturation at 95°C for 10 s, annealing at gene-specific temperatures ranging from 61.1°C to 66.1°C for 15 s, and extension at 72°C for 30 s. All samples were analysed in technical triplicate to ensure reproducibility. Primer sequences used for target and reference genes are provided in Supplementary Table S1. Relative gene expression levels were normalized against glyceraldehyde-3-phosphate dehydrogenase (GAPDH) and calculated using the comparative 2^−ΔΔ^^*Ct*^ method.

### Enzyme-linked immunosorbent assay

Collagen type I secretion in HDFs exposed to ADSC-derived secretomes was quantified using a human collagen type I alpha 1 (COL1A1) ELISA kit (ABclonal, Wuhan, China). The measured collagen levels reflect total collagen accumulation within the experimental system, including both newly synthesized fibroblast-derived collagen and ECM proteins originating from the conditioned media. Consequently, ELISA measurements represent overall collagen content rather than exclusively *de novo* collagen production by HDFs.

In parallel, inflammatory cytokine production by THP-1-derived macrophages following exposure to ADSC secretomes was assessed using human IL-10 and TNF-α ELISA kits (ABclonal, Wuhan, China). All assays were conducted in accordance with the manufacturers’ recommended procedures. OD values were recorded using a microplate reader, and protein concentrations were determined from analyte-specific standard calibration curves to ensure quantitative accuracy and reproducibility.

### Statistical analysis

Quantitative data are expressed as mean ± SD. Statistical comparisons among experimental groups were performed using one-way analysis of variance (ANOVA) followed by Tukey’s multiple-comparison *post hoc* test with OriginPro 2023 software (OriginLab, Northampton, MA, USA). Differences were considered statistically significant at *P *< 0.05.

## Results and discussion

### IronQ priming preserves ADSC viability and mesenchymal surface phenotype

**Figure 1:**
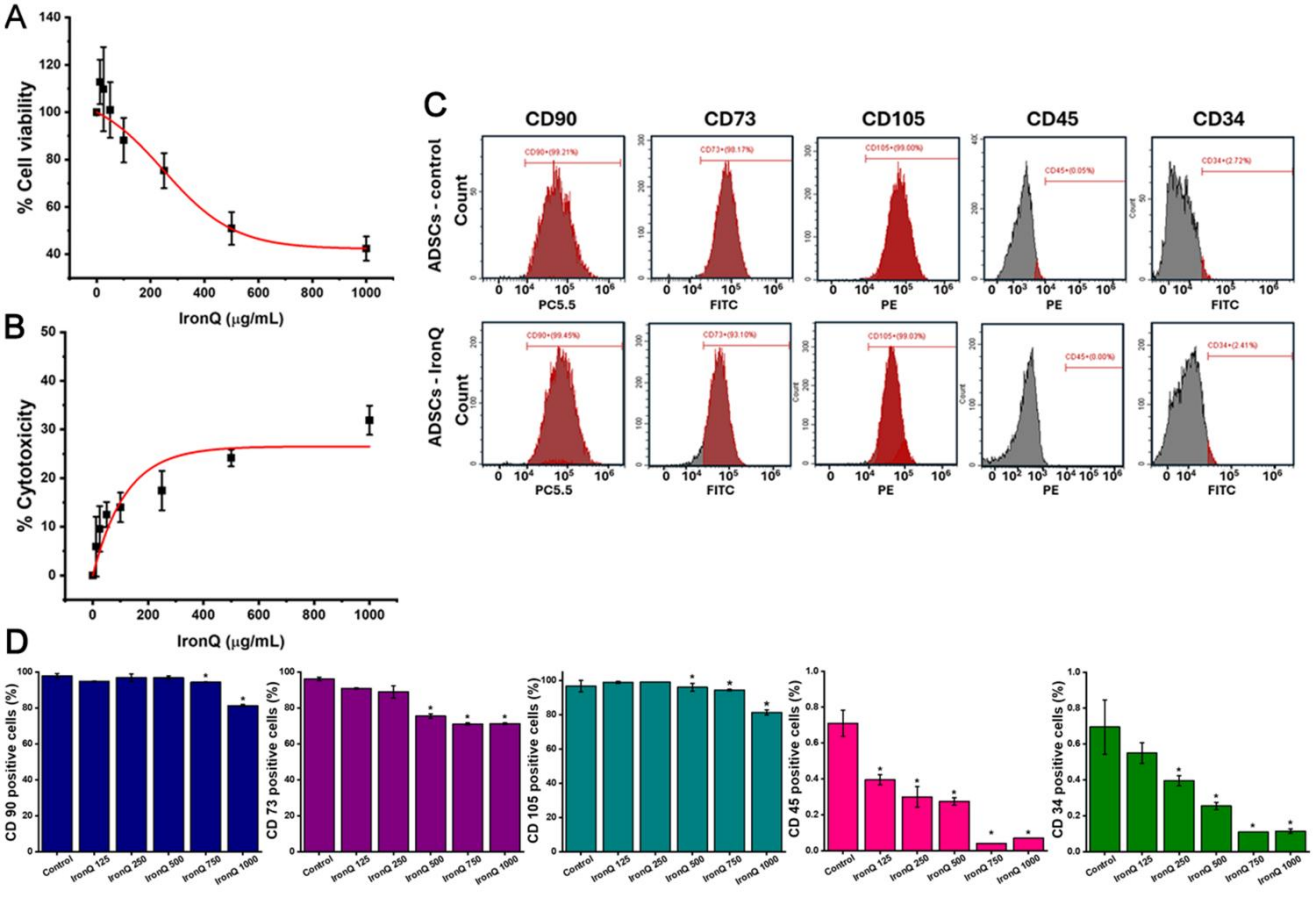
Effects of IronQ treatment on adipose-derived mesenchymal stem cells (ADSCs) viability and mesenchymal stem cell marker expression. (**A**) MTT assay of ADSCs following 24 h exposure to increasing concentrations of IronQ. (**B**) LDH release assay after IronQ treatment. (**C**) Representative flow cytometry profiles of mesenchymal stem cell markers in untreated and IronQ-treated ADSCs. (**D**) Quantification of MSC surface marker expression shown in (**C**). Results from three independent experiments are shown as mean ± SD. Statistical significance was evaluated by ANOVA with Tukey correction (*P < 0.05).

The effects of IronQ priming on ADSC viability were initially examined to determine the biocompatibility range suitable for subsequent priming experiments [32]. MTT analysis demonstrated that ADSCs maintained high metabolic activity following 24 h exposure to IronQ, with cell viability remaining above 80% at concentrations up to 250 µg mL^−1^. In contrast, higher concentrations (>500 µg mL^−1^) induced marked cytotoxicity, with viability decreasing to approximately 50% (Figure 1A). This dose-dependent decline indicates that excessive IronQ exposure exceeds the cellular tolerance threshold and may disrupt metabolic activity [33].

LDH release analysis showed a similar concentration-dependent response following IronQ exposure. ADSCs treated with 0–250 µg mL^−1^ IronQ exhibited minimal membrane damage, whereas higher concentrations produced elevated LDH release, indicating increased cellular injury (Figure 1B). Together, these complementary assays confirm that IronQ is well tolerated by ADSCs within a defined concentration window and that cytotoxic effects emerge only at supraphysiological doses. Importantly, the convergence of metabolic (MTT) and membrane integrity (LDH) data strengthens confidence in the identified safe concentration range for subsequent priming studies.

We next examined whether IronQ preconditioning altered the characteristic MSC surface phenotype, as preservation of MSC identity is essential for maintaining paracrine functionality [34]. Flow cytometric analysis demonstrated that IronQ treatment largely preserved the expression of canonical MSC markers across biologically relevant concentrations. A modest reduction in CD73 expression was observed only at the highest concentration tested (1000 µg mL^−1^), while no significant changes were detected at lower, biologically relevant concentrations (Figure 1C and D). Although significant differences in MSC marker expression were observed across the full concentration range, primarily at higher IronQ concentrations, pairwise comparison between untreated ADSCs and the optimized IronQ condition (250 µg mL^−1^) revealed no statistically significant differences in CD73, CD90 or CD105 expression. Together, these findings indicate that IronQ priming at biologically relevant and non-cytotoxic concentrations does not induce phenotypic drift or loss of mesenchymal characteristics. Consistent with the preserved surface marker profile, ADSCs retained the characteristic spindle-shaped fibroblast-like morphology of MSCs at biologically relevant non-cytotoxic IronQ concentrations, whereas higher concentrations were associated with reduced cell density and morphological alterations (Supplementary Figure S2).

Previous studies using PBMCs and MSCs have likewise demonstrated that IronQ maintains low cytotoxicity when administered at biologically compatible concentrations [23, 24, 26]. In contrast, several metal oxide-based nanoparticles have been reported to induce oxidative stress-mediated cytotoxicity, mitochondrial dysfunction and downregulation of MSC surface markers at comparable or even lower doses [35, 36]. The ability of IronQ to preserve MSC phenotype while avoiding overt cytotoxicity distinguishes it from less biocompatible inorganic nanomaterials and supports its suitability as a biochemical priming agent.

Although trilineage differentiation assays were not reevaluated following IronQ treatment, the commercially obtained ADSCs used in this study were supplier-characterized as meeting the minimal ISCT criteria. Future studies assessing the impact of IronQ on multilineage differentiation potential would further strengthen MSC characterization. Collectively, these results demonstrate that IronQ priming is cytocompatible and preserves MSC identity at concentrations up to 250 µg mL^−1^. By defining a clear therapeutic window that maintains ADSC viability and surface phenotype, this section establishes a robust foundation for subsequent investigations into IronQ uptake, transcriptomic reprogramming and secretome modulation. While IronQ priming preserved ADSC viability and canonical MSC surface marker expression, subsequent transcriptomic and secretomic analyses revealed substantial transcriptomic and secretomic reprogramming associated with altered paracrine activity.

In addition, to exclude potential bias arising from differences in cell number during CM collection, ADSC viability and cell number were quantitatively assessed at the time of CM harvest. No significant differences in cell viability or cell number were observed between control and IronQ-treated ADSCs, confirming comparable cell densities between the groups (Supplementary Figure S3). These results indicate that subsequent differences in secretome composition and downstream functional assays are unlikely to be attributed to variations in cell number but rather reflect treatment-induced qualitative modulation of ADSC paracrine activity.

### Cellular uptake of IronQ in ADSCs

**Figure 2:**
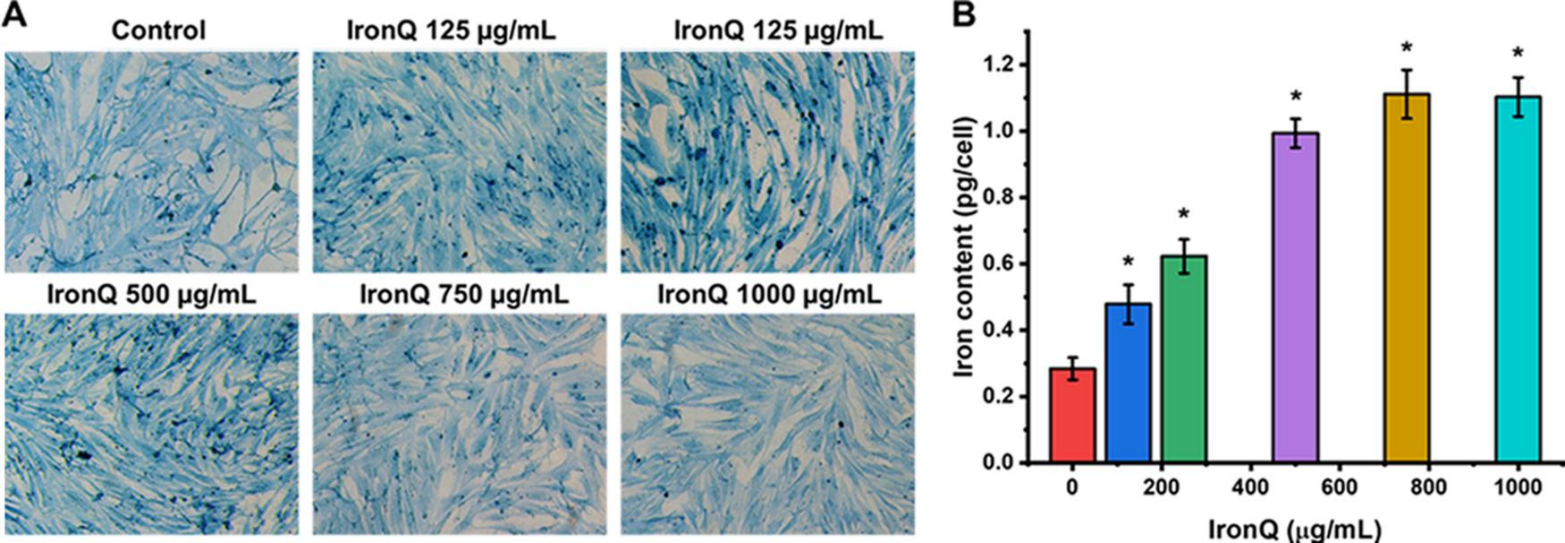
Uptake of IronQ by adipose-derived mesenchymal stem cells (ADSCs). (**A**) Prussian blue staining of ADSCs after 24 h incubation with increasing concentrations of IronQ (0–1000 µg mL^−1^). Intracellular iron deposits are visualized as blue cytoplasmic staining. Scale bars = 100 µm. Images shown are representative of three separate experiments. (**B**) Cellular iron levels measured by inductively coupled plasma-optical emission spectrometry (ICP-OES) following IronQ treatment. Values are shown as mean ± SD from three independent experiments. Statistical significance was assessed using one-way ANOVA with Tukey correction (*P < 0.05 versus untreated cells).

Based on our initial observations demonstrating that IronQ priming preserves ADSC viability and mesenchymal phenotype within a defined nontoxic concentration range, we next investigated whether IronQ is efficiently internalized by ADSCs under these priming conditions. Efficient intracellular uptake is a critical prerequisite for downstream modulation of intracellular signaling pathways and subsequent reprogramming of the MSC secretome, which underpins the functional outcomes of priming strategies [37]. Intracellular accumulation of IronQ was initially evaluated using Prussian blue staining, a classical histochemical method that detects ferric ions (Fe^3+^) through the formation of insoluble iron–ferrocyanide complexes. ADSCs were exposed to increasing concentrations of IronQ (0–1000 µg mL^−1^) for 24 h prior to staining. As shown in Figure 2A, IronQ uptake increased in a clear dose-dependent manner. Untreated control cells exhibited minimal Prussian blue staining, confirming negligible endogenous iron accumulation under baseline conditions. In contrast, IronQ-treated ADSCs displayed progressively intensified blue cytoplasmic staining with increasing IronQ concentrations, indicating enhanced intracellular iron accumulation. To quantitatively validate these observations, intracellular iron content was further measured using inductively coupled plasma-optical emission spectrometry (ICP-OES). The ICP-OES analysis confirmed a dose-dependent increase in intracellular iron accumulation following IronQ treatment. Intracellular iron levels increased progressively across the tested concentration range and reached a plateau at approximately 1.1 ± 0.07 pg Fe per cell in ADSCs treated with 750 and 1000 µg mL^−1^ IronQ (Figure 2B). These quantitative findings were consistent with the qualitative Prussian blue staining results, further confirming efficient intracellular uptake of IronQ by ADSCs.

Notably, robust intracellular staining and measurable iron accumulation were already evident at 250 µg mL^−1^, corresponding to the concentration range that maintained high cell viability and preserved canonical MSC surface marker expression (Figure 1). ICP-OES analysis revealed that ADSCs treated with the optimized priming concentration (250 µg mL^−1^) accumulated approximately 0.62 ± 0.05 pg Fe per cell, indicating that substantial intracellular accumulation could be achieved within a biologically compatible concentration range. This concordance indicates that efficient intracellular delivery of IronQ can be achieved without compromising cellular health or mesenchymal identity, a balance that is often difficult to achieve with iron-based nanomaterials [38]. At higher concentrations (>500 µg mL^−1^), more intense intracellular staining and elevated iron accumulation were observed, reflecting increased intracellular iron burden, although such concentrations fall outside the optimal priming window defined by viability and phenotypic stability. These findings suggest that intracellular iron accumulation of approximately 0.62 pg Fe per cell is sufficient to support downstream ADSC functional reprogramming without compromising mesenchymal integrity.

Importantly, Prussian blue signals were predominantly localized within the cytoplasmic compartment rather than being restricted to the cell surface, supporting genuine cellular internalization rather than passive adsorption of IronQ onto the plasma membrane. Similar intracellular localization patterns have been reported for iron-containing nanomaterials and polyphenol–metal complexes, where surface chemistry critically influences cellular trafficking and intracellular fate [39]. This cytoplasmic localization is mechanistically relevant, as intracellular iron availability enables interaction with redox-sensitive signaling pathways and intracellular regulatory networks that regulate MSC function, immunomodulatory capacity and paracrine activity [40]. In line with this concept, previous studies have demonstrated that polyphenol–metal complexes can achieve effective cytoplasmic delivery and modulate intracellular signaling without inducing nuclear damage or genomic instability [41].

Efficient intracellular delivery represents a critical determinant of successful MSC priming strategies. In this context, IronQ demonstrates a distinct advantage over many conventional nanomaterial-based systems, which often suffer from limited cellular uptake, endosomal or lysosomal sequestration, or dose-limiting cytotoxicity that restricts biological efficacy [42, 43]. The overlap between the concentration range supporting robust intracellular accumulation and that preserving ADSC viability and phenotype further underscores the favorable therapeutic window of IronQ compared with more aggressive metal or iron oxide nanoparticle formulations, which frequently induce oxidative stress, loss of stemness or DNA damage at comparable intracellular iron burdens [44]. Collectively, these findings demonstrate that ADSCs efficiently internalize IronQ in a dose-dependent manner. The alignment between efficient intracellular accumulation, preserved MSC phenotype and minimal cytotoxicity establishes a critical mechanistic foundation for subsequent intracellular reprogramming and secretome modulation. Importantly, this uptake profile distinguishes IronQ from conventional iron-based nanomaterials used for stem cell priming or imaging, positioning IronQ not merely as a passive iron carrier, but as a controllable biochemical priming agent capable of intracellular modulation without the safety liabilities commonly associated with nanoparticle-based systems.

### IronQ preconditioning induces a distinct transcriptomic program in ADSCs

**Figure 3:**
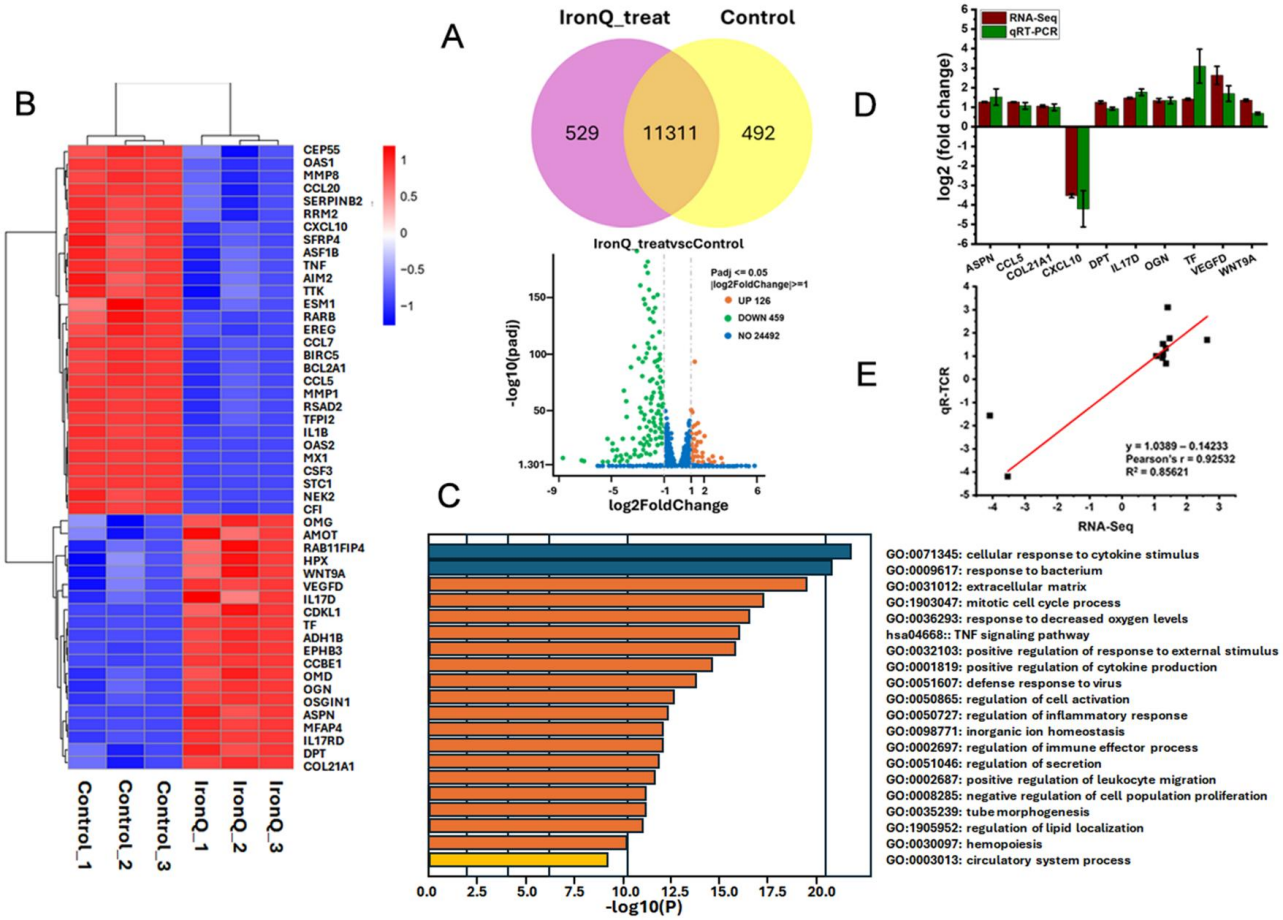
Transcriptomic profiling of IronQ-treated adipose-derived mesenchymal stem cells (ADSCs). (**A**) Venn diagram and volcano plot of differentially expressed genes (DEGs) identified between untreated and IronQ-treated ADSCs by DESeq2 analysis (|log_2_ fold change| ≥ 1, adjusted *P *≤ 0.05). (**B**) Heatmap showing expression patterns of the top 100 DEGs in control and IronQ-treated ADSCs. (**C**) Gene ontology (GO) and Kyoto encyclopedia of genes and genomes (KEGG) enrichment analyses of the top 100 DEGs following IronQ treatment. (D) qRT-PCR analysis of 10 selected DEGs identified from RNA-seq datasets. Relative expression levels were normalized to GAPDH and calculated using the comparative 2^−ΔΔ^^*Ct*^ method. Values represent log_2_ fold change relative to untreated ADSCs. (**E**) Correlation analysis between RNA-seq and qRT-PCR fold-change values. Results were obtained from three independent experiments and are presented as mean ± SD.

While iron-based materials have been widely explored for stem cell labeling and imaging applications, their capacity to selectively reprogram MSC transcriptomes toward regenerative functions remains incompletely defined. Here, we present a comprehensive transcriptomic analysis demonstrating that IronQ acts not merely as an intracellular iron source, but as a biochemical priming agent that reshapes ADSC gene expression programs linked to angiogenesis, immunomodulation and ECM remodeling. To elucidate the molecular mechanisms underlying IronQ-mediated modulation of ADSC function, comparative transcriptomic profiling of IronQ-preconditioned and untreated (basal) ADSCs was performed using RNA sequencing (RNA-seq). A total of 12 332 genes were co-expressed in both the groups, with 529 genes uniquely expressed in IronQ-treated ADSCs and 492 genes uniquely expressed in basal controls. Differential expression analysis identified 585 significantly altered genes following IronQ stimulation (adjusted *P* < 0.05, |log_2_ fold change| > 1), including 126 upregulated and 459 downregulated genes relative to basal controls (Figure 3A). The predominance of downregulated transcripts indicates that IronQ priming does not induce indiscriminate transcriptional activation, but instead promotes selective and controlled transcriptional reprogramming, potentially relieving constraints on regenerative pathways rather than triggering a generalized stress response. Consistent with this interpretation, unsupervised hierarchical clustering and heatmap visualization of the top 100 DEGs revealed a clear segregation between IronQ-treated and basal ADSCs (Figure 3B). This robust clustering pattern across biological replicates supports the conclusion that IronQ induces a reproducible and coordinated transcriptional shift, rather than stochastic or noise-driven gene expression changes. To gain functional insight into the biological processes influenced by IronQ, GO and KEGG pathway enrichment analyses were performed. Enrichment analysis presented in Figure 3C revealed prominent activation of pathways involved in ECM organization, TNF-mediated inflammatory signaling, immune modulation, iron homeostasis and morphogenic programs associated with vascular structure formation. These functional categories are central to angiogenesis, immunomodulation and tissue remodeling, providing a molecular rationale for the enhanced paracrine and pro-regenerative phenotypes observed following IronQ priming. Notably, enrichment of ECM- and morphogenesis-related pathways suggests that IronQ biases ADSCs toward a matrix-supportive and vasculogenic phenotype, rather than solely augmenting growth factor secretion.

Ten representative DEGs identified from RNA-seq analysis were subsequently examined by qRT-PCR. Figure 3D and E demonstrates strong agreement between the two analytical approaches in both expression direction and relative fold-change magnitude. At a mechanistic level, the IronQ-induced transcriptomic landscape reflects coordinated modulation of pathways governing inflammation, ECM remodeling, angiogenesis and iron metabolism. This pattern aligns with established MSC priming paradigms, in which controlled environmental cues bias stem cell function toward regenerative outcomes. For instance, hypoxic preconditioning activates HIF-1α-dependent gene programs, enriching VEGF-dominant secretomes and accelerating tissue repair in ischemic and diabetic models [45, 46]. Similarly, inflammatory cytokine licensing with IFN-γ and/or TNF-α promotes immunomodulatory MSC phenotypes via upregulation of IDO, PGE_2_ and TNF signaling pathways, thereby enhancing macrophage crosstalk and inflammation resolution [47]. Compared with these biologically driven priming strategies, nanomaterial-based priming offers advantages in tunability, stability and scalability. Previous studies have shown that iron-based nanomaterials can modulate intracellular redox balance, iron homeostasis and stress-responsive signaling, leading to selective transcriptomic reprogramming rather than global transcriptional activation [48]. The transcriptional profile induced by IronQ extends beyond angiogenesis alone, encompassing immune regulation and ECM remodeling pathways, consistent with emerging evidence that iron-mediated intracellular cues fine-tune MSC plasticity through redox and metabolic regulation [49]. Collectively, these transcriptomic analyses demonstrate that IronQ treatment reshaped ADSC gene expression toward a regeneration-oriented molecular phenotype. Functional enrichment analyses highlighted coordinated activation of pathways related to ECM dynamics, angiogenic regulation, immune-associated signaling and iron homeostasis, providing mechanistic insight into how intracellular IronQ uptake may influence downstream paracrine activity. By reshaping the ADSC transcriptome in a controlled and nonrandom manner, IronQ emerges as a distinct biochemical priming agent capable of biasing MSCs toward wound-relevant regenerative programs, effectively bridging intracellular modulation with therapeutic secretome enhancement.

### Composition of the secretome derived from IronQ-preconditioned ADSCs

**Figure 4:**
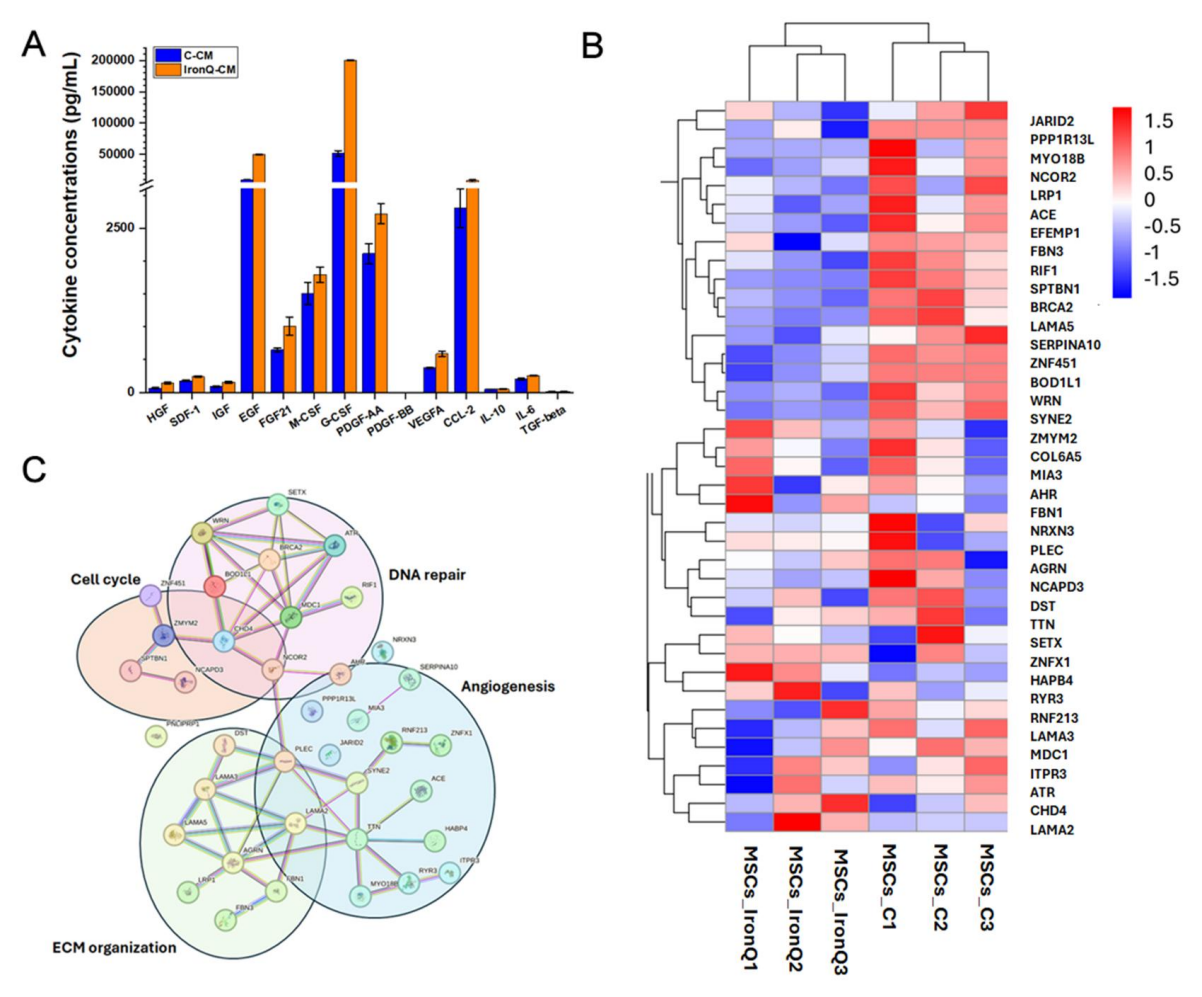
Analysis of soluble factors and secretome-associated proteins in IronQ-preconditioned ADSCs. (**A**) Luminex-based quantification of soluble cytokines and growth factors in conditioned media collected from untreated ADSCs (C-CM) and IronQ-treated ADSCs (IronQ-CM). Cytokine concentrations are expressed as pg mL^−1^. *P < 0.05 versus C-CM. (**B**) Heatmap of 66 proteins identified in ADSC-derived secretomes by LC–MS/MS analysis. (**C**) STRING-based protein–protein interaction network generated from differentially expressed secretome-associated proteins. Data were derived from three independent experiments and are reported as mean ± SD. Abbreviations: EGF, epidermal growth factor; FGF-2, fibroblast growth factor-2; HGF, hepatocyte growth factor; IL-10, interleukin-10; MCP-1 (CCL-2), monocyte chemoattractant protein-1; PDGF-AA, platelet-derived growth factor-AA; PDGF-AB/BB, platelet-derived growth factor-AB/BB; VEGF-A, vascular endothelial growth factor-A; SDF-1, stromal cell-derived factor-1; IGF, insulin-like growth factor; G-CSF, granulocyte colony-stimulating factor; M-CSF, macrophage colony-stimulating factor.

All conditioned media were collected from ADSCs with comparable viability and cell number, as established in Section ‘IronQ priming preserves ADSC viability and mesenchymal surface phenotype’, thereby minimizing potential bias arising from differences in cell density. To determine whether the intracellular uptake and transcriptomic reprogramming induced by IronQ (Sections ‘Cellular uptake of IronQ in ADSCs’ and ‘IronQ preconditioning induces a distinct transcriptomic program in ADSCs’) translate into functional alterations in paracrine signaling, we next examined the composition of the ADSC secretome following IronQ preconditioning. Conditioned media collected from untreated ADSCs (C-CM) and IronQ-preconditioned ADSCs (IronQ-CM) were analysed, with a focus on soluble mediators involved in cell proliferation, migration, angiogenesis, immune regulation and ECM remodeling, which are central to wound repair and tissue regeneration. Targeted cytokine and growth factor profiling was first performed to quantify 14 representative paracrine mediators associated with regenerative activity. All analytes were detectable in both C-CM and IronQ-CM, indicating that IronQ preconditioning does not induce aberrant or ectopic factor secretion, but rather modulates the abundance of physiologically relevant secreted molecules. Temporal changes in individual analytes are shown in Figure 4A. Overall, IronQ preconditioning resulted in a global increase in secretory output, consistent with a transcriptionally primed and functionally active paracrine phenotype. Several growth factors with established roles in wound healing and angiogenesis—including FGF-2, HGF, macrophage colony-stimulating factor (M-CSF), granulocyte colony-stimulating factor (G-CSF), platelet-derived growth factor-AA (PDGF-AA), transforming growth factor-β (TGF-β) and VEGF-A—were significantly elevated in IronQ-CM compared with controls. In parallel, chemokines and immunomodulatory factors, such as CC chemokine ligand 2 (CCL-2; MCP-1), interleukin-10 (IL-10), interleukin-6 (IL-6) and stromal cell-derived factor-1α (SDF-1α), were consistently increased following IronQ priming. Among the quantified analytes, G-CSF, PDGF-AA, EGF and CCL-2 were present at the highest concentrations, indicating their dominant contribution to the IronQ-modulated ADSC secretome. In contrast, PDGF-BB remained low or near the detection limit, consistent with previous reports describing limited baseline PDGF-BB secretion by ADSCs [50].

The magnitude and breadth of cytokine enhancement observed in IronQ-CM compare favorably with established MSC priming strategies. Hypoxic preconditioning has been shown to increase VEGF-A, HGF and SDF-1α primarily through HIF-1α-dependent pathways but often exerts limited effects on immunomodulatory cytokines such as IL-10 [51]. Conversely, inflammatory cytokine licensing with IFN-γ and/or TNF-α robustly enhances immunoregulatory mediators (e.g. IDO, PGE_2_, IL-10) but frequently suppresses growth factor secretion and may compromise long-term MSC viability or promote senescence [52]. In contrast, IronQ preconditioning simultaneously augments angiogenic, immunomodulatory and chemoattractant factors without evidence of phenotypic instability or cytotoxic stress, suggesting a more balanced and multifunctional secretome profile. The concurrent elevation of G-CSF, PDGF-AA, FGF-2, EGF, VEGF-A, HGF and SDF-1α and CCL-2 is biologically meaningful, as these factors act cooperatively to promote endothelial sprouting, recruitment of perivascular support cells and macrophage-mediated tissue remodeling—key processes underlying effective wound healing rather than fibrotic repair [53, 54]. The accompanying increase in IL-10 further supports a shift toward inflammation resolution, addressing a recognized limitation of cytokine-licensed MSC secretomes that may prolong inflammatory signaling.

To obtain an unbiased, system-level view of secretome remodeling, label-free quantitative proteomic analysis of C-CM and IronQ-CM was performed using LC–MS/MS (*n* = 3). A total of 66 proteins were consistently detected across both conditions (Supplementary Table S2). Hierarchical clustering and heatmap analysis revealed distinct and coordinated expression patterns between C-CM and IronQ-CM (Figure 4B), indicating structured remodeling rather than random variation in protein abundance. PPI analysis using the STRING database demonstrated strong interaction enrichment among differentially expressed proteins (PPI enrichment *P* < 1.0 × 10^−16^; average local clustering coefficient = 0.606), consistent with a functionally coherent secretome network. Network clustering further resolved these proteins into four major functional clusters (Figure 4C). Cluster I was dominated by ECM structural proteins, including multiple collagen subunits, fibronectin and proteoglycans, which are essential for scaffold formation, cell adhesion and matrix remodeling during tissue repair. Cluster II comprised proteins related to cell cycle regulation, while Cluster III included proteins involved in DNA repair and cellular stress responses, consistent with the cytoprotective functions attributed to MSC secretomes. Cluster IV contained proteins associated with angiogenic regulation, reinforcing the pro-vascular signature identified in the targeted cytokine analysis. Previous proteomic studies of MSC secretomes generated under hypoxic or inflammatory priming frequently report enrichment of either angiogenic or immunomodulatory protein subsets, often accompanied by stress-associated signatures indicative of harsh priming conditions [55]. In contrast, IronQ-CM exhibited coordinated enrichment of ECM, angiogenic, and cytoprotective protein clusters without excessive activation of stress-related pathways, supporting the notion that IronQ induces controlled functional reprogramming rather than damage-associated secretion. Collectively, targeted cytokine profiling and global proteomic analyses demonstrate that IronQ preconditioning induces both quantitative and qualitative remodeling of the ADSC secretome. IronQ-CM is enriched in a coordinated network of angiogenic growth factors (VEGF-A, HGF, FGF-2, PDGF-AA), immunomodulatory cytokines (G-CSF, CCL-2 and IL-10), chemoattractants (SDF-1α) and ECM-associated proteins, forming a functionally integrated secretome optimized for wound repair rather than isolated pathway activation. Compared with hypoxic or inflammatory priming strategies, IronQ generates a broader and more balanced secretory profile that supports vascularization, immune resolution and matrix remodeling. This integrated secretome remodeling provides a mechanistic link between intracellular IronQ-mediated reprogramming and enhanced regenerative performance observed in subsequent functional assays, supporting IronQ as a practical and clinically relevant priming strategy for MSC-based regenerative therapies.

### Secretome from IronQ-preconditioned ADSCs enhances endothelial proliferation and angiogenic activity *in vitro*

Vascular regeneration during wound healing depends on coordinated endothelial cell expansion, directional migration, structural organization and vessel maturation, collectively supporting tissue repair and restoration [56]. Building on the intracellular uptake and transcriptomic reprogramming induced by IronQ preconditioning (Sections ‘Cellular uptake of IronQ in ADSCs’ and ‘IronQ preconditioning induces a distinct transcriptomic program in ADSCs’), as well as the coordinated remodeling of the ADSC secretome (Section ‘Composition of the secretome derived from IronQ-preconditioned ADSCs’), we next investigated whether these molecular changes translate into functional pro-angiogenic activity at the cellular level.

**Figure 5:**
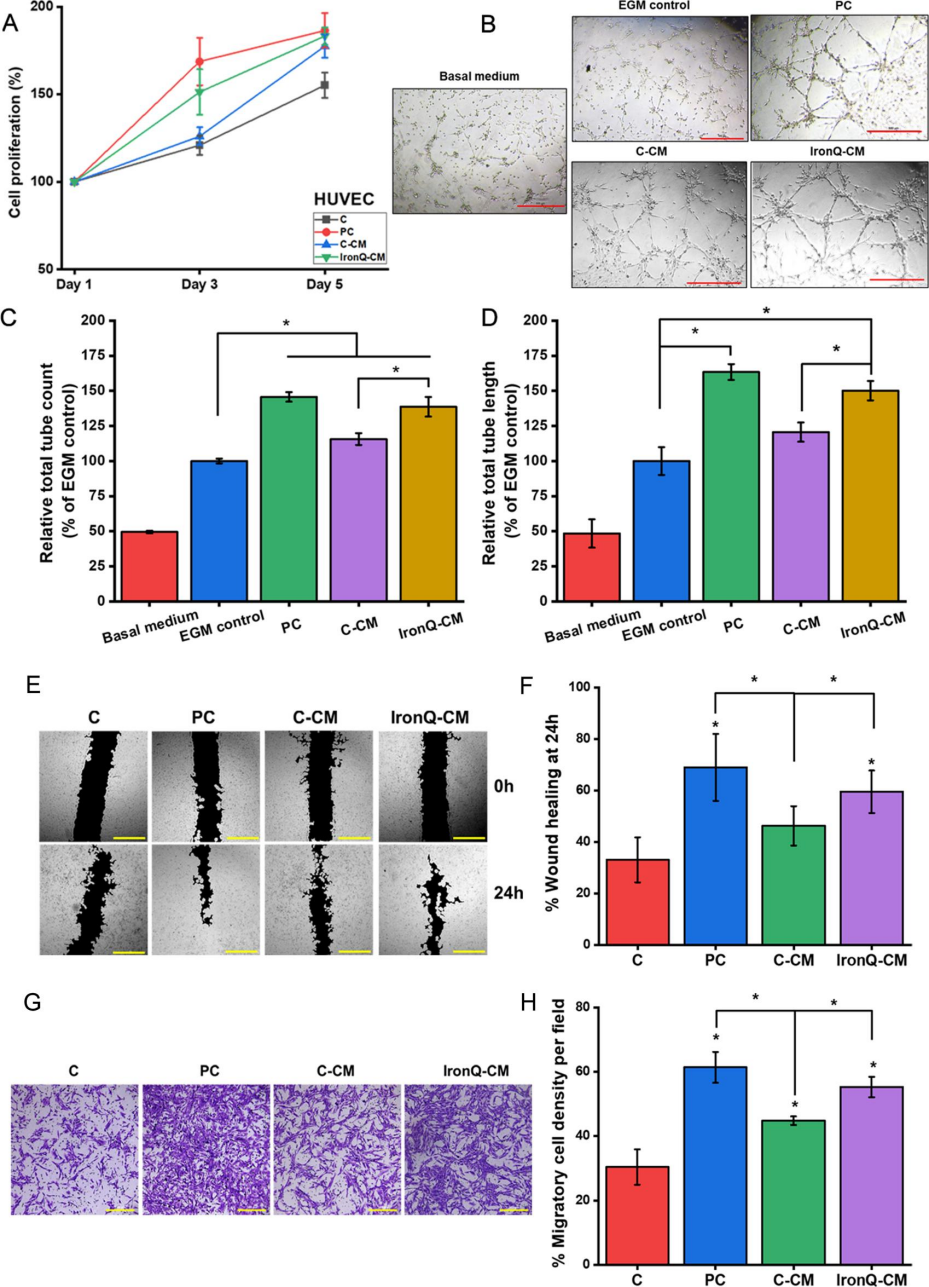
Effects of IronQ-preconditioned ADSC secretomes on endothelial proliferation, angiogenic network formation and migration. (**A**) CCK-8 analysis of HUVEC proliferation following treatment with conditioned media from untreated ADSCs (C-CM) or IronQ-preconditioned ADSCs (IronQ-CM) at 24, 72 and 120 h. (**B**) Representative micrographs of endothelial tube formation under basal medium, 50% endothelial growth medium (EGM control), positive control (100% EGM), C-CM and IronQ-CM conditions. Scale bar = 500 µm. (**C, D**) Quantification of tube number and total tube length from the networks shown in (**B**) using WimTube software. (**E, F**) Representative images and quantitative analysis of HUVEC migration evaluated by scratch wound assay at 0 and 24 h. (**G, H**) Transwell migration assay and corresponding quantification of migrated HUVECs under the indicated treatment conditions. Scale bar = 300 µm. Results are expressed as mean ± SD from three independent experiments. Statistical significance was determined using one-way ANOVA with Tukey’s *post hoc* test (*P < 0.05).

Endothelial proliferation was first evaluated using a CCK-8 assay in HUVECs exposed to conditioned media derived from untreated ADSCs (C-CM), IronQ-preconditioned ADSCs (IronQ-CM) or basal medium alone. As illustrated in Figure 5A, CM derived from IronQ-preconditioned ADSCs (IronQ-CM) promoted significantly greater HUVEC proliferation than both C-CM and basal medium controls throughout the experimental period. Compared with secretomes from untreated ADSCs, IronQ-CM produced a more pronounced proliferative response, suggesting that IronQ priming enhances the pro-angiogenic activity of the ADSC secretome. This observation is consistent with the increased abundance of angiogenesis-associated soluble factors detected in IronQ-CM, including VEGF-A, HGF and FGF-2 (Section ‘Composition of the secretome derived from IronQ-preconditioned ADSCs’), which are known to support endothelial growth and vascular regenerative responses [57].

To further assess angiogenic functionality beyond proliferation, endothelial tube formation assays were performed on Matrigel. Although 50% EGM (control) established a defined baseline level of angiogenic activity, both C-CM and IronQ-CM significantly enhanced tube formation beyond the EGM-defined baseline condition. In contrast, basal medium alone exhibited minimal tube formation, confirming that the angiogenic response was driven by bioactive factors present in the ADSC secretome. Importantly, IronQ-CM induced a markedly greater increase in total tube length (151.12 ± 6.9% relative to the EGM control) compared with C-CM (120.58 ± 6.86%), accompanied by increased branching points and overall network complexity (Figure 5B–D). These findings indicate that IronQ preconditioning enhances not only endothelial activation but also the structural organization of vascular networks. Notably, despite the presence of VEGF and bFGF in EGM, IronQ-CM further augmented angiogenesis beyond this pre-stimulated baseline, indicating that its effects are not redundant with exogenous growth factor supplementation. Mechanistically, while VEGF primarily drives endothelial proliferation and sprouting via VEGFR2 signaling, factors enriched in IronQ-CM—such as HGF, PDGF-AA and SDF-1α—likely activate complementary signaling pathways involved in endothelial migration, stabilization and network maturation [58, 59]. The integration of these signaling axes may explain the formation of more highly interconnected and functionally organized vascular structures observed in the IronQ-CM group.

To determine whether IronQ-CM also enhances additional steps of angiogenesis, endothelial cell migration was evaluated using both wound healing and Transwell migration assays. In the wound healing assay, HUVECs treated with IronQ-CM exhibited significantly accelerated wound closure at *24 *h compared with basal medium and C-CM (Figure *5*E and F), indicating enhanced collective cell migration. Consistently, Transwell migration analysis demonstrated a significantly higher number of migrated endothelial cells in the IronQ-CM group compared with all control conditions (Figure *5*G and H). Notably, the migratory response induced by IronQ-CM exceeded that observed with C-CM and approached or exceeded the effect observed in the EGM positive control, suggesting that IronQ preconditioning enhances the secretion of factors that actively promote endothelial chemotaxis. This enhanced migratory capacity is mechanistically supported by the enrichment of chemotactic and motogenic factors within IronQ-CM. In particular, the HGF/c-Met axis promotes endothelial cell motility through activation of Mitogen-activated protein kinase (MAPK) and Phosphoinositide 3-kinase (PI3K) signaling pathways, while the SDF-1α/CXCR4 axis plays a central role in directional cell migration and vascular recruitment [59, 60]. The concurrent presence of these factors likely establishes a chemotactic microenvironment that facilitates endothelial migration and vascular sprouting. Importantly, this mechanism is distinct from the VEGF-driven proliferative response provided by EGM, indicating that IronQ-CM complements rather than redundantly amplify classical angiogenic signaling. The integration of VEGF-mediated proliferation with HGF- and SDF-1α-driven migration may therefore explain the superior angiogenic performance observed in the IronQ-CM-treated groups, even under conditions where baseline growth factor stimulation is already present. Collectively, these findings demonstrate that IronQ-CM enhances multiple coordinated steps of angiogenesis, including endothelial proliferation, migration and network formation, thereby providing comprehensive functional evidence of its pro-vascular activity.

When contextualized within existing MSC priming strategies, the angiogenic efficacy of IronQ-CM is particularly notable. While hypoxic preconditioning predominantly enhances VEGF-driven angiogenesis and inflammatory cytokine licensing often shifts MSC function toward immunomodulation [55, 61], IronQ-CM induces a more balanced secretome integrating mitogenic (VEGF-A, FGF-*2*), morphogenic (HGF, PDGF-AA) and chemotactic (SDF-*1*α) factors. This coordinated profile supports not only endothelial activation but also subsequent vessel organization and stabilization. This integrated angiogenic response is particularly relevant in the context of chronic and diabetic wounds, where impaired endothelial responsiveness, insufficient VEGF signaling and defective neovascularization are major barriers to tissue repair [62]. The ability of IronQ-CM to enhance multiple steps of angiogenesis suggests that it may overcome key deficiencies associated with these conditions. Collectively, these findings position IronQ preconditioning as a promising strategy for generating functionally optimized MSC secretomes for regenerative applications.

### Secretome from IronQ-preconditioned ADSCs enhances migratory and invasive capacities of dermal fibroblasts

**Figure 6:**
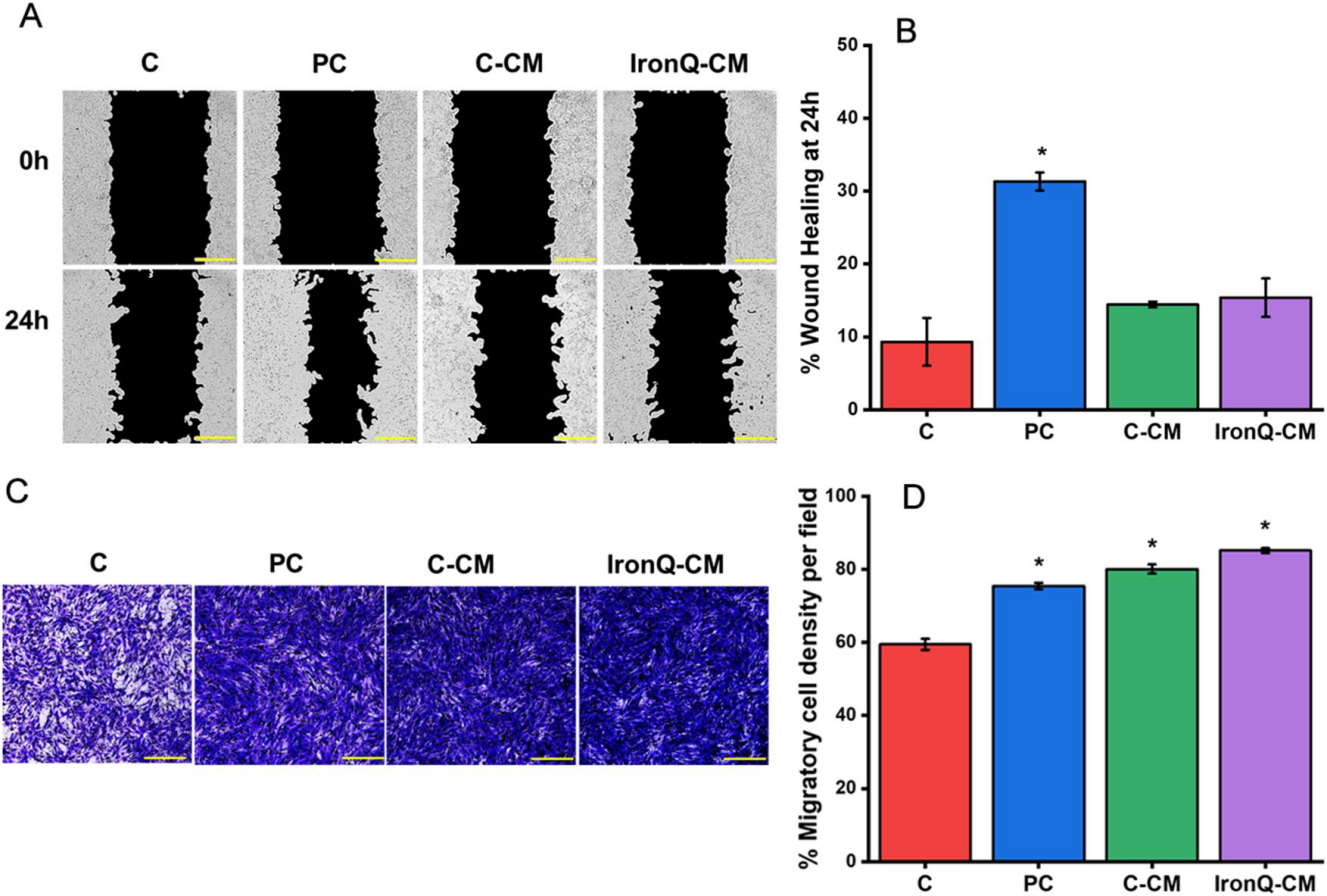
Effects of IronQ-preconditioned ADSC secretomes on fibroblast migration. (**A**) Scratch wound assay of fibroblasts treated with basal medium, conditioned medium from untreated ADSCs (C-CM) or conditioned medium from IronQ-preconditioned ADSCs (IronQ-CM). (**B**) Relative wound closure measured from the assay shown in (**A**). (**C**) Transwell migration assay of fibroblasts under the indicated treatment conditions. (**D**) Quantification of migrated fibroblasts from panel (**C**). Scale bars = 500 µm. Measurements are shown as mean ± SD from three independent experiments (*P < 0.05).

Directed migration and invasion of dermal fibroblasts are critical events during cutaneous wound healing, contributing to granulation tissue formation, ECM deposition and wound contraction [63]. Given the coordinated remodeling of the ADSC secretome induced by IronQ preconditioning (Sections ‘IronQ preconditioning induces a distinct transcriptomic program in ADSCs’ and ‘Composition of the secretome derived from IronQ-preconditioned ADSCs’) and its demonstrated pro-angiogenic activity (Section ‘Secretome from IronQ-preconditioned ADSCs enhances endothelial proliferation and angiogenic activity *in vitro*’), we next investigated whether IronQ-mediated priming enhances the ability of ADSC-derived secretomes to support fibroblast-driven repair processes. Scratch wound analysis demonstrated enhanced fibroblast migration following treatment with both C-CM and IronQ-CM compared with basal medium controls (Figure 6A and B), supporting the pro-regenerative activity of ADSC-derived secretomes [64]. Notably, fibroblasts treated with IronQ-CM displayed greater wound closure than those exposed to C-CM, suggesting that IronQ priming enhances the migration-supportive properties of the ADSC secretome. The accelerated closure kinetics suggest enhanced directional migration and cytoskeletal reorganization, processes essential for effective wound contraction and matrix deposition.

To further assess fibroblast-migratory behavior relevant to wound repair and to confirm the findings from the scratch wound healing assay, Transwell migration assays were performed. Both C-CM and IronQ-CM significantly increased the number of fibroblasts migrating through the porous membrane compared with basal control conditions (Figure 6C and D), demonstrating that ADSC-derived paracrine factors enhance directional fibroblast migration. Notably, IronQ-CM induced a significantly greater number of migrated fibroblasts than C-CM, indicating a superior capacity to stimulate fibroblast motility. These results are consistent with the enhanced wound closure observed in the scratch assay and further indicate that secretomes from IronQ-preconditioned ADSCs facilitate fibroblast migration during tissue repair [65]. The observed functional enhancement is mechanistically consistent with the enriched secretory profile of IronQ-CM. Elevated levels of HGF, FGF-2, PDGF-AA and SDF-1α—all detected at higher concentrations following IronQ priming—are well documented to promote fibroblast chemotaxis, actin cytoskeletal remodeling and invasive behavior during tissue repair [66–68]. In addition, increased IL-10 levels may indirectly support fibroblast migration and matrix remodeling by attenuating excessive inflammatory signaling, thereby creating a permissive microenvironment for productive wound healing rather than fibrotic repair [69].

When compared with established MSC priming strategies, the fibroblast-migratory effects of IronQ-CM are particularly notable. Hypoxia-primed MSC secretomes have been reported to enhance fibroblast migration primarily through VEGF- and HGF-mediated pathways, but often exhibit limited ECM-modulatory signaling [70]. Inflammatory licensing with IFN-γ or TNF-α can promote fibroblast recruitment indirectly via immune cell crosstalk; however, prolonged inflammatory priming frequently biases MSCs toward immunosuppressive phenotypes and may increase the risk of impaired matrix deposition or fibrotic responses [71]. In contrast, IronQ-CM integrates fibroblast-activating growth factors with chemotactic and matrix-remodeling cues, supporting productive fibroblast migration and invasion without excessive inflammatory stimulation. Such secretome characteristics may be beneficial in chronic wound environments, including diabetic ulcers, where impaired fibroblast recruitment, defective cell migration and abnormal ECM remodeling contribute to delayed tissue regeneration [72]. By enhancing both migratory and invasive capacities of dermal fibroblasts, IronQ-tuned ADSC secretomes may help overcome these pathological constraints. Collectively, these findings demonstrate that the ADSC secretome robustly promotes dermal fibroblast migration, and that IronQ preconditioning further potentiates this pro-migratory effect. IronQ-CM enhances directional fibroblast migration more effectively than unprimed secretomes, as evidenced by accelerated wound closure in scratch assays and increased transmembrane migration in Transwell assays. This enhanced migratory response is likely mediated by the coordinated upregulation of fibroblast-activating growth factors and chemotactic mediators, including HGF, FGF-2, PDGF-AA and SDF-1α, together with anti-inflammatory factors that create a permissive repair environment. Compared with conventional hypoxic or inflammatory priming approaches, IronQ generates a more balanced pro-migratory secretome that supports fibroblast-driven granulation tissue formation while minimizing excessive inflammatory or fibrotic signaling. Such balanced regulation may be advantageous for minimizing excessive fibroblast activation and pathological scar formation during tissue remodeling. These results further reinforce the therapeutic potential of IronQ-tuned ADSC secretomes as a cell-free strategy to accelerate dermal repair and functional wound regeneration.

### Secretome from IronQ-preconditioned ADSCs promotes proliferation and activation of dermal fibroblasts

**Figure 7:**
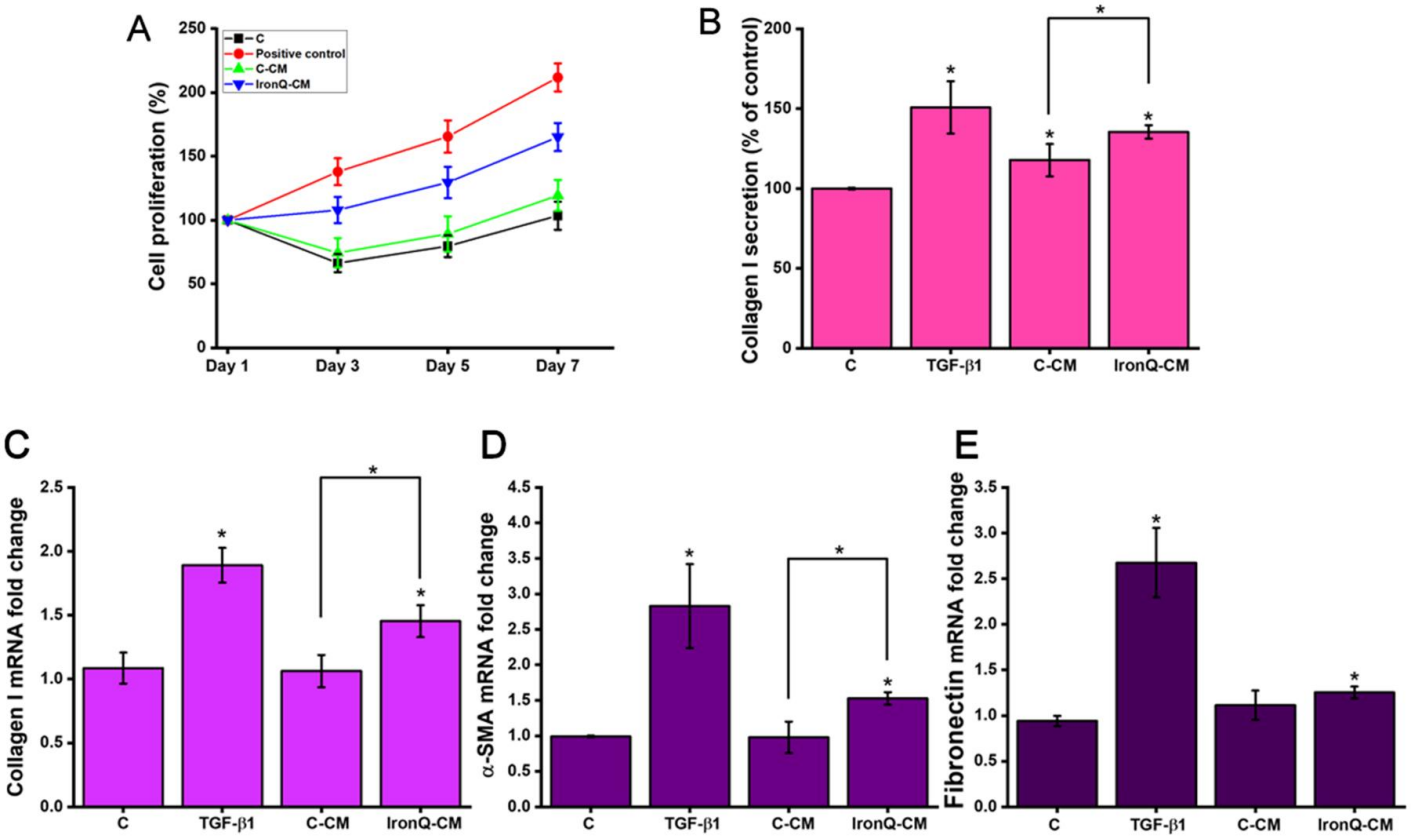
Secretome derived from IronQ-preconditioned ADSCs enhances fibroblast proliferation and activation-associated responses. (**A**) Proliferation of dermal fibroblasts following exposure to conditioned medium from IronQ-preconditioned ADSCs (IronQ-CM), evaluated using the CCK-8 assay at days 1, 3, 5, and 7. Quantitative results are expressed as mean ± standard deviation (SD) from three independent experiments. *p < 0.05 versus corresponding control groups. (**B**) Collagen type I (Col-I) levels in fibroblast culture supernatants following treatment with ADSC-derived conditioned media, determined by ELISA. Data are shown as mean ± SD from three independent experiments. (**C–E**) Relative mRNA expression of fibroblast activation-associated genes, including *COL1A1* (**C**), *ACTA2* (encoding α-smooth muscle actin; α-SMA) (**D**), and fibronectin *FN1* (**E**) determined by quantitative real-time PCR (qRT-PCR). Gene expression levels were normalized to GAPDH and calculated using the comparative 2^−ΔΔ^^*Ct*^ method. Results represent mean ± SD from three independent experiments.

Fibroblast proliferation and activation are central to granulation tissue formation, ECM deposition and wound contraction during cutaneous repair [73]. To determine whether IronQ-mediated priming enhances the capacity of the ADSC secretome to support fibroblast-driven repair, HDFs were treated with basal medium (DMEM), CM from untreated ADSCs (C-CM), CM from IronQ-preconditioned ADSCs (IronQ-CM), or a positive control (10% FBS). Fibroblast proliferation was first assessed using a CCK-8 assay. As shown in Figure 7A, IronQ-CM significantly increased HDF proliferation compared with both DMEM and C-CM, indicating that IronQ preconditioning enhances the mitogenic potency of the ADSC secretome rather than merely preserving baseline trophic activity.

The proliferative response induced by IronQ-CM approached, but did not exceed, that observed with 10% FBS, suggesting physiological activation rather than supraphysiological overstimulation. This increase in fibroblast proliferation is consistent with the elevated levels of growth factors detected in IronQ-CM, including HGF, FGF-2 and PDGF-AA (Section ‘Composition of the secretome derived from IronQ-preconditioned ADSCs’), all of which are well established to promote fibroblast survival and cell-cycle progression during wound repair [74]. Because fibroblast activation is tightly coupled to ECM synthesis, we next quantified collagen type I (Col-I) production at the protein level. As shown in Figure 7B, HDFs treated with IronQ-CM exhibited significantly higher levels of Col-I compared with the DMEM and C-CM groups. However, as supported by proteomic analysis (Figure 4B), ADSC-conditioned media inherently contain ECM proteins. Therefore, ELISA-based measurements should be interpreted as reflecting total collagen accumulation within the co-culture system rather than exclusively *de novo* collagen synthesis by HDFs. This finding suggests an enhanced matrix-enriched microenvironment associated with fibroblast activation and ECM remodeling. Notably, this increase in collagen production occurred in the absence of exogenous TGF-β supplementation, suggesting that IronQ-CM supports ECM synthesis through endogenous paracrine cues rather than direct profibrotic stimulation [75]. Gene expression analysis further demonstrated that fibroblasts treated with IronQ-CM exhibited elevated mRNA levels of COL1A1, α-SMA and FN1 compared with the DMEM and C-CM groups (Figure 7C–E), indicating enhanced activation-associated transcriptional responses. These genes are canonical markers of activated fibroblasts and early myofibroblast differentiation, processes required for wound contraction and ECM remodeling [76]. Importantly, the coordinated upregulation of ECM components and cytoskeletal markers suggests a functional activation state rather than terminal fibroblast differentiation. Together, these findings indicate that the enhanced collagen deposition observed in the IronQ-CM group likely reflects both exogenous ECM contribution and active fibroblast-associated ECM synthesis, with transcriptional evidence supporting genuine cellular activation. A limitation of this study is that ELISA-based collagen measurements may include contributions from ECM proteins present in ADSC-CM and should therefore be interpreted as total collagen accumulation within the system. Future studies incorporating baseline subtraction using cell-free CM, protein-normalized conditions or fibroblast-specific collagen tracing approaches would further strengthen quantitative interpretation. Although fibroblast activation is essential for ECM deposition and wound closure, excessive or persistent fibroblast activation may contribute to pathological fibrosis and scar formation. Therefore, maintaining a balance between regenerative ECM remodeling and uncontrolled fibroproliferative responses is a critical consideration in wound-healing therapies [77]. In the present study, IronQ-CM promoted fibroblast proliferation, migration and ECM-associated gene expression under controlled *in vitro* conditions without exogenous TGF-β stimulation, suggesting a controlled reparative activation profile rather than uncontrolled fibroproliferative signaling. Moreover, the concurrent enrichment of immunomodulatory mediators such as IL-10 and reparative growth factors including HGF may support coordinated tissue remodeling while limiting excessive profibrotic signaling. Nevertheless, long-term *in vivo* studies will be required to determine whether sustained exposure to IronQ-modulated secretomes influences fibrotic remodeling or scar formation during later stages of wound healing.

Compared with established MSC priming strategies, IronQ-CM exhibits a distinct and more balanced fibroblast-activating profile. Inflammatory cytokine licensing (e.g. IFN-γ or TNF-α) enhances fibroblast activation indirectly via inflammatory signaling and is often associated with elevated TGF-β activity and prolonged α-SMA expression, increasing the risk of excessive fibrosis [78]. In contrast, IronQ-CM promotes fibroblast proliferation and activation through coordinated upregulation of HGF, FGF-2, PDGF-AA and SDF-1α, together with IL-10 and other immunomodulatory mediators, supporting productive wound repair rather than pathological fibrosis [79]. Consistent with this profile, enrichment of HGF—both known to support reversible fibroblast activation and matrix synthesis while limiting excessive TGF-β-driven fibrosis—suggests that IronQ-CM favors a reparative, remodeling-competent fibroblast phenotype [80]. Collectively, these results demonstrate that IronQ-preconditioned ADSC secretomes enhance dermal fibroblast proliferation and activation, as evidenced by increased cell growth, collagen type I production and coordinated upregulation of key ECM and cytoskeletal genes. Compared with conventional hypoxic or inflammatory priming approaches, IronQ-CM promotes a balanced fibroblast activation state that supports granulation tissue formation and wound contraction without excessive profibrotic signaling. Together with the enhanced angiogenic and immunomodulatory effects described in preceding sections, these findings position IronQ-tuned ADSC secretomes as a multifunctional, cell-free therapeutic strategy for coordinated and effective wound repair.

The proliferative response induced by IronQ-CM approached, but did not exceed, that observed with 10% FBS, suggesting physiological activation rather than supraphysiological overstimulation. This increase in fibroblast proliferation is consistent with the elevated levels of growth factors detected in IronQ-CM, including HGF, FGF-2, and PDGF-AA (Section ‘Composition of the secretome derived from IronQ-preconditioned ADsCs’), all of which are well established to promote fibroblast survival and cell-cycle progression during wound repair [74]. Because fibroblast activation is tightly coupled to ECM synthesis, we next quantified collagen type I (Col-I) production at the protein level. As shown in Figure 7B, HDFs treated with IronQ-CM exhibited significantly higher levels of Col-I compared with the DMEM and C-CM groups. However, as supported by proteomic analysis (Figure 4B), ADSC-conditioned media inherently contain ECM proteins. Therefore, ELISA-based measurements should be interpreted as reflecting total collagen accumulation within the co-culture system rather than exclusively *de novo* collagen synthesis by HDFs. This finding suggests an enhanced matrix-enriched microenvironment associated with fibroblast activation and ECM remodeling. Notably, this increase in collagen production occurred in the absence of exogenous TGF-β supplementation, suggesting that IronQ-CM supports ECM synthesis through endogenous paracrine cues rather than direct profibrotic stimulation [75]. Gene expression analysis further demonstrated that fibroblasts treated with IronQ-CM exhibited elevated mRNA levels of COL1A1, α-SMA and FN1 compared with DMEM and C-CM groups (Figure 7C–E), indicating enhanced activation-associated transcriptional responses. These genes are canonical markers of activated fibroblasts and early myofibroblast differentiation, processes required for wound contraction and ECM remodeling [76]. Importantly, the coordinated upregulation of ECM components and cytoskeletal markers suggests a functional activation state rather than terminal fibroblast differentiation. Together, these findings indicate that the enhanced collagen deposition observed in the IronQ-CM group likely reflects both exogenous ECM contribution and active fibroblast-associated ECM synthesis, with transcriptional evidence supporting genuine cellular activation. A limitation of this study is that ELISA-based collagen measurements may include contributions from ECM proteins present in ADSC-CM and should therefore be interpreted as total collagen accumulation within the system. Future studies incorporating baseline subtraction using cell-free CM, protein-normalized conditions or fibroblast-specific collagen tracing approaches would further strengthen quantitative interpretation. Although fibroblast activation is essential for ECM deposition and wound closure, excessive or persistent fibroblast activation may contribute to pathological fibrosis and scar formation. Therefore, maintaining a balance between regenerative ECM remodeling and uncontrolled fibroproliferative responses is a critical consideration in wound-healing therapies [77]. In the present study, IronQ-CM promoted fibroblast proliferation, migration and ECM-associated gene expression under controlled *in vitro* conditions without exogenous TGF-β stimulation, suggesting a controlled reparative activation profile rather than uncontrolled fibroproliferative signaling. Moreover, the concurrent enrichment of immunomodulatory mediators such as IL-10 and reparative growth factors including HGF may support coordinated tissue remodeling while limiting excessive profibrotic signaling. Nevertheless, long-term *in vivo* studies will be required to determine whether sustained exposure to IronQ-modulated secretomes influences fibrotic remodeling or scar formation during later stages of wound healing.

Compared with established MSC priming strategies, IronQ-CM exhibits a distinct and more balanced fibroblast-activating profile. Inflammatory cytokine licensing (e.g. IFN-γ or TNF-α) enhances fibroblast activation indirectly via inflammatory signaling and is often associated with elevated TGF-β activity and prolonged α-SMA expression, increasing the risk of excessive fibrosis [78]. In contrast, IronQ-CM promotes fibroblast proliferation and activation through coordinated upregulation of HGF, FGF-2, PDGF-AA and SDF-1α, together with IL-10 and other immunomodulatory mediators, supporting productive wound repair rather than pathological fibrosis [79]. Consistent with this profile, enrichment of HGF—both known to support reversible fibroblast activation and matrix synthesis while limiting excessive TGF-β–driven fibrosis—suggests that IronQ-CM favors a reparative, remodeling-competent fibroblast phenotype [80]. Collectively, these results demonstrate that IronQ-preconditioned ADSC secretomes enhance dermal fibroblast proliferation and activation, as evidenced by increased cell growth, collagen type I production and coordinated upregulation of key ECM and cytoskeletal genes. Compared with conventional hypoxic or inflammatory priming approaches, IronQ-CM promotes a balanced fibroblast activation state that supports granulation tissue formation and wound contraction without excessive profibrotic signaling. Together with the enhanced angiogenic and immunomodulatory effects described in preceding sections, these findings position IronQ-tuned ADSC secretomes as a multifunctional, cell-free therapeutic strategy for coordinated and effective wound repair.

### Secretome from IronQ-preconditioned ADSCs promotes macrophage polarization toward the M2 phenotype

**Figure 8:**
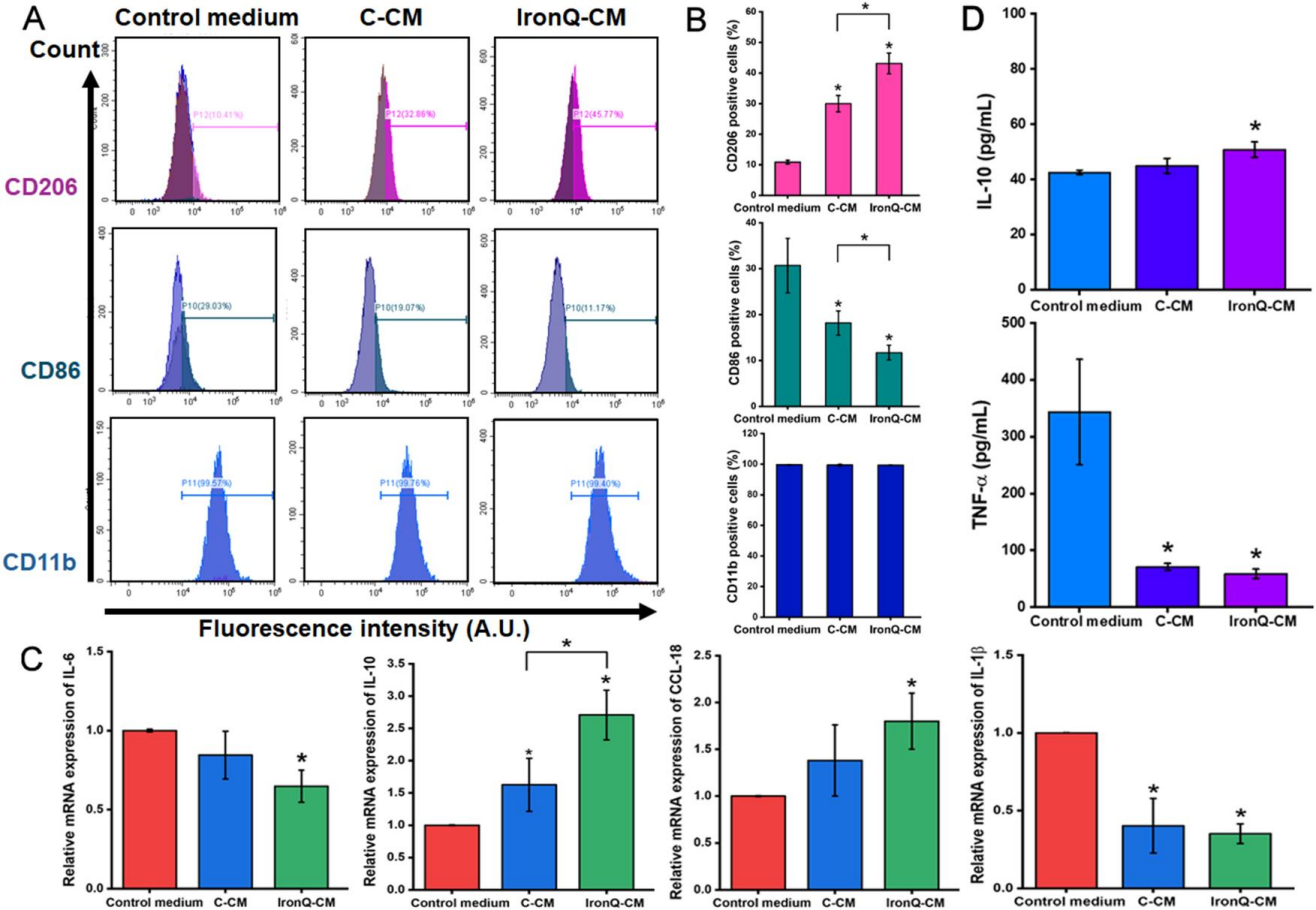
Secretome from IronQ-preconditioned ADSCs modulates macrophage polarization toward a reparative M2-like phenotype. (**A**) Representative flow cytometry histograms showing surface expression of CD206 (M2-associated marker), CD86 (M1-associated marker) and CD11b (pan-macrophage marker) in THP-1-derived macrophages treated with control medium, conditioned medium from untreated ADSCs (C-CM), or conditioned medium from IronQ-preconditioned ADSCs (IronQ-CM). (**B**) Quantitative flow cytometry analysis demonstrating an increased proportion of CD206^+^ macrophages and a reduced proportion of CD86^+^ macrophages following IronQ-CM treatment, while CD11b expression remained comparable across groups. (**C**) Relative mRNA expression levels of M1-associated cytokines (IL-1β, IL-6) and M2-associated markers (CCL-18, IL-10) in macrophages treated with C-CM or IronQ-CM, as determined by qRT-PCR. Gene expression was normalized to a housekeeping gene (GAPDH) and expressed relative to control conditions. (**D**) Cytokine levels in macrophage culture supernatants measured by ELISA, showing decreased TNF-α and increased IL-10 secretion following IronQ-CM treatment compared with control and C-CM groups. Data are presented as mean ± SD from three independent experiments. *P < 0.05 versus control or C-CM.

Macrophages are key regulators of inflammation resolution, angiogenesis and tissue remodeling during wound healing through their remarkable phenotypic plasticity [81]. To determine whether IronQ-mediated priming enhances the immunomodulatory capacity of the ADSC secretome, we examined the effects of conditioned media derived from IronQ-preconditioned ADSCs (IronQ-CM) on macrophage polarization *in vitro*. Flow cytometric analysis of THP-1-derived macrophages demonstrated that exposure to IronQ-CM significantly increased the proportion of CD206^+^ macrophages, a canonical marker of the reparative M2 phenotype, compared with both C-CM and basal control conditions. Concomitantly, the proportion of CD86^+^ macrophages, indicative of the pro-inflammatory M1 phenotype, was markedly reduced following IronQ-CM treatment (Figure 8A and B). Importantly, expression of the pan-macrophage marker CD11b remained unchanged across all conditions, indicating that IronQ-CM selectively modulates macrophage polarization rather than affecting macrophage viability or differentiation status. These findings indicate that IronQ-CM actively biases macrophages toward a reparative phenotype instead of inducing nonspecific immunosuppression.

Consistent with the phenotypic shift observed at the surface marker level, transcriptional analysis revealed that IronQ-CM significantly suppressed the expression of the M1-associated cytokine IL-1β, while markedly upregulating M2-associated genes, including CCL-18 and IL-10 (Figure 8C). Notably, IL-6 expression exhibited context-dependent modulation rather than uniform suppression. Although IL-6 is traditionally categorized as a pro-inflammatory cytokine, accumulating evidence suggests that IL-6 can support alternative macrophage activation, metabolic adaptation, and tissue repair under regenerative conditions, highlighting its pleiotropic role in wound healing environments [82, 83]. The selective modulation of inflammatory gene expression observed here further supports the notion that IronQ-CM induces a functionally regulated M2-like phenotype rather than a binary M1-to-M2 switch. Protein-level analysis by ELISA corroborated these transcriptional findings. Macrophages treated with IronQ-CM exhibited a pronounced reduction in TNF-α secretion, a hallmark M1 cytokine associated with chronic inflammation and tissue damage, alongside a significant increase in IL-10 production (Figure 8D). IL-10 is a central mediator of inflammation resolution and has been shown to promote angiogenesis, fibroblast activation and ECM remodeling during wound repair [84, 85]. The coordinated suppression of TNF-α and enhancement of IL-10 therefore reflects a functional immunoregulatory phenotype conducive to tissue regeneration. Compared with commonly used MSC priming strategies, IronQ-CM appears to elicit a more coordinated immunomodulatory profile. Whereas cytokine-licensed MSC secretomes (e.g. IFN-γ/TNF-α) predominantly induce immunosuppressive pathways with limited support for angiogenic or matrix-remodeling processes, and hypoxia-primed MSCs show variable M2-polarizing effects [55], IronQ-CM concurrently suppresses M1-associated cytokines while enhancing IL-10- and CCL-18-related reparative signaling. The enrichment of IL-10, CCL-2, VEGF-A and HGF in IronQ-CM may contribute to this effect by promoting alternative macrophage activation and coordinated crosstalk with endothelial cells and fibroblasts during inflammation resolution [86]. Together, these features suggest that IronQ priming may more closely mimic physiological immune transitions observed in effective wound healing.

The observed cytokine profile and macrophage responses suggest that IronQ-primed ADSC secretomes support the induction of anti-inflammatory, M2-like macrophage characteristics via secreted regulatory factors. By suppressing pro-inflammatory M1 markers while enhancing IL-10- and CCL-18-associated anti-inflammatory programs, IronQ-CM fosters an immune microenvironment that supports angiogenesis, fibroblast activation and matrix remodeling. The combined immunomodulatory and reparative responses observed in this study suggest that IronQ-primed ADSC secretomes may provide therapeutic benefit in chronic wound environments characterized by persistent inflammation and defective tissue repair.

## Conclusion

This study demonstrates that IronQ preconditioning induces coordinated transcriptomic and secretomic reprogramming in ADSCs, resulting in a multifunctional paracrine profile relevant to wound repair. IronQ-preconditioned ADSCs exhibit enhanced secretion of angiogenic growth factors, fibroblast-regulatory mediators, immunomodulatory cytokines and ECM-associated proteins, collectively addressing key pathological features of chronic wounds, including impaired angiogenesis, dysregulated stromal activity and unresolved inflammation. Consistent with these molecular changes, the IronQ-modulated secretome promotes endothelial proliferation and angiogenic network formation, enhances fibroblast migration and matrix production, and biases macrophage polarization toward a reparative M2-like phenotype. By integrating nanomaterial-based intracellular priming with a secretome-focused therapeutic approach, this work proposes a cell-free regenerative strategy that may overcome several limitations associated with direct cell transplantation, including donor variability, storage constraints and regulatory complexity. Compared with biologically driven priming methods, IronQ enables controlled modulation of MSC function while preserving cell viability and phenotype, yielding a balanced and functionally coherent secretome. In addition, the intrinsic magnetic resonance visibility of IronQ may support future development of image-guided priming or delivery platforms. Despite these findings, the current study is limited to *in vitro* experimental systems, and further evaluation in clinically relevant *in vivo* wound models will be necessary to determine the therapeutic potential and long-term stability of IronQ-modulated secretomes. Further mechanistic studies will also be necessary to delineate causal pathways and optimize priming parameters. In addition, scalable manufacturing and delivery strategies must be established to support translational applications. Although conditioned media were collected from ADSCs with comparable cell viability and cell number, normalization based on total protein content or cell number was not performed. Therefore, minor variations in secretome concentration cannot be completely excluded. Future studies incorporating quantitative normalization strategies would further strengthen the interpretation of secretome-specific effects.

In summary, IronQ preconditioning represents a tunable and scalable approach to direct MSC secretome composition toward coordinated angiogenic, stromal and immunomodulatory functions. These findings support the potential of IronQ-modulated secretomes as a bioactive, cell-free platform for regenerative wound therapy.

## Supplementary Material

rbag132_Supplementary_Data
