# Supplementary material for: Iron–quercetin nanocomplex preconditioning reprograms the mesenchymal stem cell secretome to drive angiogenic, fibroblast and immunoregulatory wound repair
Source: Regen Biomater. 2026 Jun 15;13:rbag132. doi: 10.1093/rb/rbag132 (PMC13355599; doi:10.1093/rb/rbag132)
Supplement: rbag132_Supplementary_Data [file rbag132_supplementary_data.docx]

**Iron–Quercetin Nanocomplex Preconditioning Reprograms the Mesenchymal Stem Cell Secretome to Drive Angiogenic, Fibroblast, and Immunoregulatory Wound Repair**

Jiraporn Kantapan^1,2^, Phattarawadee Innuan^1,2^, Donraporn Daranarong^3,4^, Sittiruk Roytrakul^5^, Krit Jaikumkao ^2^, Gwenaël Rolin^6,7^, Céline Viennet-Steiner ^6^, Worapong Khaodee^8^, Padchanee Sangthong^9^, Kittichai Wantanajittikul^2^, Winita Punyodom^4,9^, Nathupakorn Dechsupa ^1,2 *^

^1^ Molecular Imaging and Therapy Research Unit, Department of Radiologic Technology, Faculty of Associated Medical Sciences, Chiang Mai University, Chiang Mai 50200, Thailand.

^2^ Department of Radiologic Technology, Faculty of Associated Medical Sciences, Chiang Mai University, Chiang Mai 50200, Thailand.

^3^ Multidisciplinary Research Institute, Chiang Mai University, Chiang Mai 50200, Thailand

^4^ Center of Excellence in Materials Science and Technology, Chiang Mai University, Chiang Mai 50200, Thailand

^5^ National Center for Genetic Engineering and Biotechnology, National Science and Technology Development Agency, Pathum Thani 12120, Thailand

^6^ INSERM CIC-1431, CHU Besançon, F-25000 Besançon, France.

^7^ Université Marie et Louis Pasteur, UM RIGHT, F-25000 Besançon, France.

^8^ Faculty of Allied Health Sciences, Burapha University, Chonburi, 20131, Thailand

^9^ Department of Chemistry, Faculty of Science, Chiang Mai University, Chiang Mai, 50200, Thailand.

* Corresponding authors

Nathupakorn Dechsupa, Tel.: +66-53939412, e-mail address: [nathupakorn.d@cmu.ac.th](mailto:nathupakorn.d@cmu.ac.th)

**Table S1**

List of primers used for quantitative real‐time PCR.

| **Gene** | **Forward primer (5′–3′)** | **Reverse primer (5′–3′)** |
| --- | --- | --- |
| *ASPN* | CCT TCA CAC ATC GCA CTG | CTT CTT GGC TCT CTT GTT GG |
| *CCL-5* | GGA GAT GAG CTA GGA TGG AG | GTA ACT GCT GCT GTG TGG TAG |
| *CCL18* | CCA GAA ATA CAT CAG CGA CC | GCA GCT CAA CAA TAG AAA TC |
| *CXCL-10* | CAC GTG TTG AGA TCA TTG CTA C | GAA GCA CTG CAT CGA TTT TG |
| *COL21A1* | GTG ACT ACC CTG TGC TGG AG | GAA ATC GTG AGG ACT TGG C |
| *DPT* | CAG ACA ATG GAA CTA CGC C | GAA GTA GCG GCT CTG GAA TC |
| *IL-1B* | GTA CGA TCA CTG AAC TGC AC | CAA CAC GCA GGA CAG GTA CAG |
| *IL-6* | CAT CAC TGG TCT TTT GGA G | CTG CGC AGA ATG AGA TGA G |
| *IL-10* | CTG AGC TTC TCT GTG AAC GA | CAG CTA GAA AGC GTG GTC AG |
| *IL-17D* | GCC AGC TAA GAG TTC CAA AG | CAA TAC CTG CTT GGA TTC TTC |
| *OGN* | GAT AAA GCC AGC ACC ACC | GTG GGC ATT TCA TCA TTT TC |
| *TF* | GGA TGC AGG TTT GGT GTA TG | CTT CTT CAC CAC AGC AAC AG |
| *VEGFD* | CTC TAG CTG CCT GAT GTC AAC | CAC TGG TCC ATG TTC ATT ACT G |
| *WNT9A* | CTG CGG AGA CAA CCT TAA GTA C | CTC ATA CTT GTG CTT CAG ATG C |
| *Col I* | CTG GTG ATG CTG GTG CTA AAG | GAC CTT TGC CGC CTT CTT TGC |
| *ACTA2* | CAA TGA GCT TCG TGT TGC CC | GGC ATA GAG AGA CAG CAC CG |
| *FN1* | CCG CCG AAT GTA GGA CAA GA | AGG GTT CTT CAT CAG TGC CA |
| *GAPDH* | GTA TCG TGG GAA GGA CTC ATG AC | GAA CAT CAT CCC TGC CTC TAC |

**Table S2**

List of 66 proteins consistently detected in C-CM and IronQ-CM, identified by LC–MS/MS-based proteomic analysis of ADSC secretomes.

| Protein_ID | Gene | Comparison | logFC | Statistic | P.Value | adj.P.Val |
| --- | --- | --- | --- | --- | --- | --- |
| Q96K12 | FAR2 | C2D_vs_IQ2D | -0.514261403 | -21.38326162 | 3.01E-05 | 0.001985587 |
| Q9H799 | CPLANE1 | C2D_vs_IQ2D | -0.553997432 | -7.672317102 | 0.001920788 | 0.063386002 |
| Q9UFH2 | DNAH17 | C2D_vs_IQ2D | 0.329191286 | 5.758214045 | 0.007095308 | 0.156096779 |
| Q9Y4E5 | ZNF451 | C2D_vs_IQ2D | -0.095917843 | -6.432969212 | 0.014847471 | 0.181815756 |
| Q8WXH0 | SYNE2 | C2D_vs_IQ2D | -0.822206736 | -6.474838972 | 0.018251725 | 0.181815756 |
| Q8NFC6 | BOD1L1 | C2D_vs_IQ2D | -0.753486155 | -7.223597298 | 0.018267421 | 0.181815756 |
| Q14191 | WRN | C2D_vs_IQ2D | -0.273371377 | -4.77431075 | 0.025506933 | 0.181815756 |
| Q5UIP0 | RIF1 | C2D_vs_IQ2D | -0.165920866 | -3.489345434 | 0.025576082 | 0.181815756 |
| P51587 | BRCA2 | C2D_vs_IQ2D | -0.491754131 | -4.529100177 | 0.025681305 | 0.181815756 |
| Q01082 | SPTBN1 | C2D_vs_IQ2D | -0.27656076 | -5.577131491 | 0.027547842 | 0.181815756 |
| Q6UXH9 | PAMR1 | C2D_vs_IQ2D | -0.260414118 | -5.254918016 | 0.033805201 | 0.202831204 |
| Q7Z5J4 | RAI1 | C2D_vs_IQ2D | -0.286834154 | -4.101752907 | 0.042645419 | 0.228121293 |
| Q12805 | EFEMP1 | C2D_vs_IQ2D | -0.07972373 | -3.107669834 | 0.04755506 | 0.228121293 |
| Q9UK55 | SERPINA10 | C2D_vs_IQ2D | -0.101494791 | -2.882338285 | 0.051492527 | 0.228121293 |
| O15230 | LAMA5 | C2D_vs_IQ2D | -0.378072009 | -3.869264512 | 0.05528101 | 0.228121293 |
| Q9Y6Y1 | CAMTA1 | C2D_vs_IQ2D | -0.627803064 | -3.929643314 | 0.055302132 | 0.228121293 |
| Q7Z7M0 | MEGF8 | C2D_vs_IQ2D | -0.252950101 | -2.927703934 | 0.064584017 | 0.250737948 |
| Q9NWH7 | SPATA6 | C2D_vs_IQ2D | -0.185752107 | -2.596476071 | 0.072775232 | 0.262226221 |
| Q8WUF5 | PPP1R13L | C2D_vs_IQ2D | -0.532419402 | -3.42376839 | 0.075489367 | 0.262226221 |
| Q96K76 | USP47 | C2D_vs_IQ2D | -0.425644216 | -3.09460625 | 0.089483087 | 0.276312587 |
| Q9H672 | ASB7 | C2D_vs_IQ2D | -0.255995022 | -2.955560821 | 0.090904875 | 0.276312587 |
| Q9Y618 | NCOR2 | C2D_vs_IQ2D | -0.140027736 | -2.615057012 | 0.092104196 | 0.276312587 |
| P12821 | ACE | C2D_vs_IQ2D | -0.232671008 | -2.320599552 | 0.105969977 | 0.297890297 |
| P28715 | ERCC5 | C2D_vs_IQ2D | -0.149338498 | -2.45210492 | 0.111014806 | 0.297890297 |
| Q5JVS0 | HABP4 | C2D_vs_IQ2D | 0.487608336 | 2.528963962 | 0.11504083 | 0.297890297 |
| Q9NRL2 | BAZ1A | C2D_vs_IQ2D | -0.298193731 | -2.152894056 | 0.12252176 | 0.297890297 |
| Q15751 | HERC1 | C2D_vs_IQ2D | 0.207818115 | 2.427111835 | 0.124812882 | 0.297890297 |
| Q6P2Q9 | PRPF8 | C2D_vs_IQ2D | 0.206850731 | 1.978924956 | 0.126377702 | 0.297890297 |
| P08686 | CYP21A2 | C2D_vs_IQ2D | -0.247187365 | -2.208416752 | 0.144710493 | 0.322688337 |
| Q92833 | JARID2 | C2D_vs_IQ2D | -0.1465785 | -1.797895665 | 0.146676517 | 0.322688337 |
| Q75N90 | FBN3 | C2D_vs_IQ2D | -0.124607343 | -2.086103822 | 0.164484847 | 0.350193545 |
| Q16787 | LAMA3 | C2D_vs_IQ2D | -0.195866983 | -1.620837838 | 0.182085315 | 0.375550962 |
| Q07954 | LRP1 | C2D_vs_IQ2D | -0.244878983 | -1.766565201 | 0.195709418 | 0.382227361 |
| Q7Z3J2 | VPS35L | C2D_vs_IQ2D | -0.081811842 | -1.549950385 | 0.199143618 | 0.382227361 |
| Q8IUG5 | MYO18B | C2D_vs_IQ2D | -0.274638853 | -1.868084647 | 0.202696328 | 0.382227361 |
| Q14839 | CHD4 | C2D_vs_IQ2D | 0.151460702 | 1.213970527 | 0.293445822 | 0.529889914 |
| P35555 | FBN1 | C2D_vs_IQ2D | 0.33412929 | 1.331416156 | 0.297059497 | 0.529889914 |
| Q14676 | MDC1 | C2D_vs_IQ2D | -0.073417219 | -1.270946632 | 0.305351957 | 0.530348136 |
| Q03001 | DST | C2D_vs_IQ2D | -0.234634311 | -1.139476135 | 0.322674496 | 0.537753998 |
| P42695 | NCAPD3 | C2D_vs_IQ2D | -0.198090329 | -1.256610932 | 0.331980565 | 0.537753998 |
| Q9P2E3 | ZNFX1 | C2D_vs_IQ2D | 0.067499892 | 1.261158776 | 0.334059302 | 0.537753998 |
| P24043 | LAMA2 | C2D_vs_IQ2D | 0.123784693 | 1.119284824 | 0.379165605 | 0.595831665 |
| Q9UKJ3 | GPATCH8 | C2D_vs_IQ2D | 0.268269679 | 1.052275711 | 0.401681827 | 0.616534897 |
| Q8WYK1 | CNTNAP5 | C2D_vs_IQ2D | -0.148085418 | -0.882270346 | 0.436403378 | 0.645687938 |
| Q13535 | ATR | C2D_vs_IQ2D | -0.087571332 | -0.931053845 | 0.442151968 | 0.645687938 |
| Q9UBW7 | ZMYM2 | C2D_vs_IQ2D | 0.159113283 | 0.837322849 | 0.450024926 | 0.645687938 |
| Q8IVF4 | DNAH10 | C2D_vs_IQ2D | -0.314769419 | -0.855095164 | 0.473039869 | 0.660120329 |
| P17040 | ZSCAN20 | C2D_vs_IQ2D | 0.057658803 | 0.820840855 | 0.48791738 | 0.660120329 |
| Q3V6T2 | CCDC88A | C2D_vs_IQ2D | 0.131819296 | 0.771082016 | 0.490089335 | 0.660120329 |
| Q8WZ42 | TTN | C2D_vs_IQ2D | -0.117785639 | -0.744905571 | 0.503799892 | 0.665015857 |
| Q5VZP5 | STYXL2 | C2D_vs_IQ2D | -0.063143051 | -0.637199676 | 0.579161366 | 0.749502944 |
| Q9Y4C0 | NRXN3 | C2D_vs_IQ2D | -0.033894722 | -0.577199777 | 0.621579027 | 0.788927226 |
| Q15413 | RYR3 | C2D_vs_IQ2D | 0.147237772 | 0.522538874 | 0.644133718 | 0.7931466 |
| O95622 | ADCY5 | C2D_vs_IQ2D | 0.20399792 | 0.528450525 | 0.648938127 | 0.7931466 |
| Q63HN8 | RNF213 | C2D_vs_IQ2D | -0.041000442 | -0.48413418 | 0.67121951 | 0.805463412 |
| Q96PQ0 | SORCS2 | C2D_vs_IQ2D | -0.065371946 | -0.430196134 | 0.691923202 | 0.815480916 |
| O75970 | MPDZ | C2D_vs_IQ2D | -0.0308218 | -0.407341636 | 0.717718176 | 0.831042098 |
| Q9HCK8 | CHD8 | C2D_vs_IQ2D | 0.024103452 | 0.364306588 | 0.73936051 | 0.836975918 |
| A0A1B0GV03 | GOLGA6L7 | C2D_vs_IQ2D | 0.060279044 | 0.346269759 | 0.748205745 | 0.836975918 |
| A8TX70 | COL6A5 | C2D_vs_IQ2D | -0.027023754 | -0.312794553 | 0.773092731 | 0.850402004 |
| Q14573 | ITPR3 | C2D_vs_IQ2D | -0.04640695 | -0.274678058 | 0.797772403 | 0.863163584 |
| O00468 | AGRN | C2D_vs_IQ2D | -0.012803434 | -0.163239638 | 0.883819941 | 0.940840582 |
| Q7Z333 | SETX | C2D_vs_IQ2D | -0.00901688 | -0.124469948 | 0.910649208 | 0.952759305 |
| P35869 | AHR | C2D_vs_IQ2D | 0.015073882 | 0.084559945 | 0.938103577 | 0.952759305 |
| Q15149 | PLEC | C2D_vs_IQ2D | 0.017376483 | 0.083954463 | 0.940689454 | 0.952759305 |
| Q5JRA6 | MIA3 | C2D_vs_IQ2D | -0.010417584 | -0.063043267 | 0.952759305 | 0.952759305 |

**
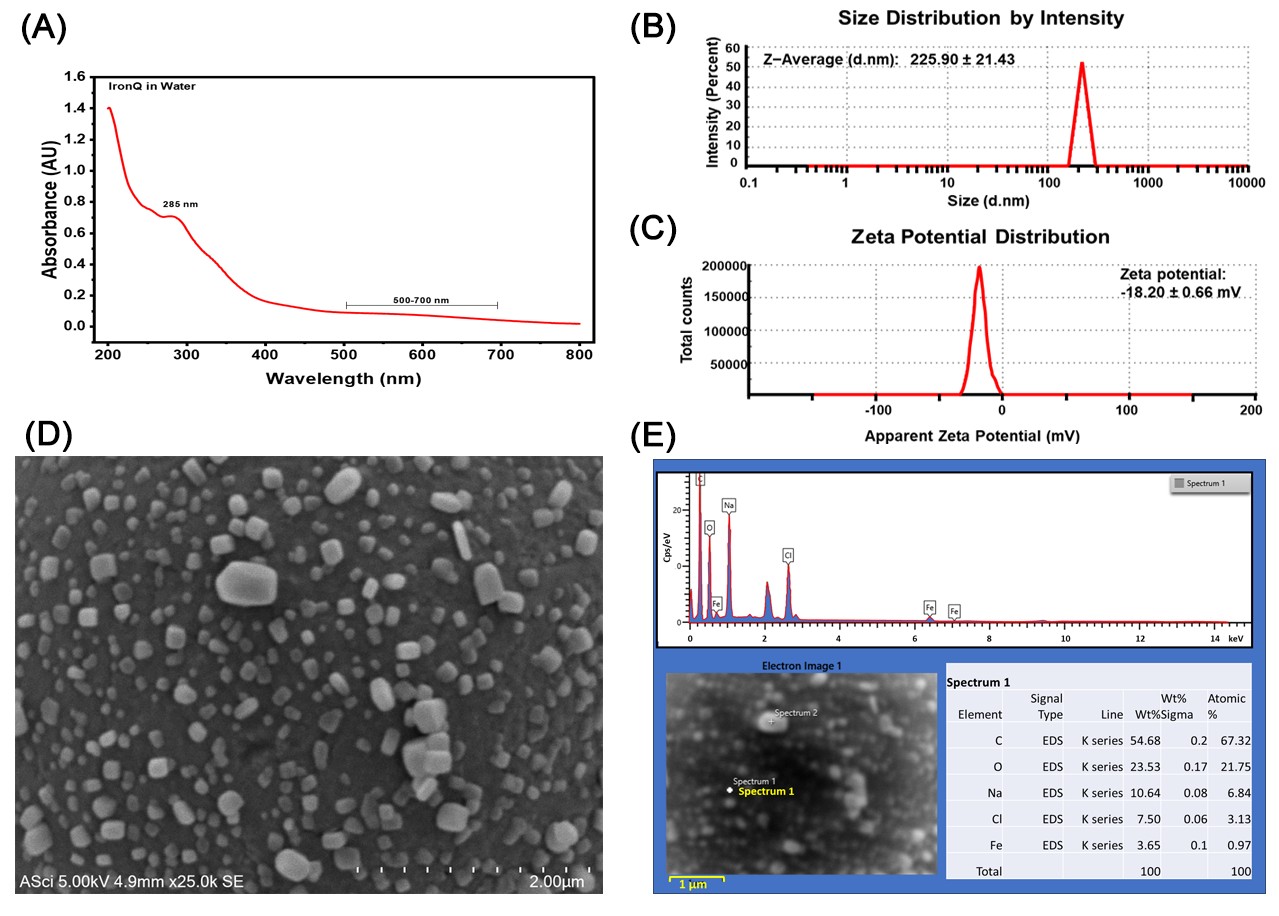
**

**Supplementary Figure S1**. Physicochemical characterization of the iron–quercetin complex (IronQ). (A) UV–visible absorption spectrum of IronQ. (B) Hydrodynamic size distribution of IronQ measured by dynamic light scattering (DLS). (C) Zeta potential analysis of IronQ. (D) Scanning electron microscopy (SEM) images of IronQ at 25,000× magnification, showing nanoscale particles with irregular amorphous morphology together with sodium chloride (NaCl) crystalline structures. (E) Energy-dispersive X-ray (EDX) spectrum of IronQ confirming the presence of major constituent elements, including carbon (C), oxygen (O), sodium (Na), chlorine (Cl), and iron (Fe).


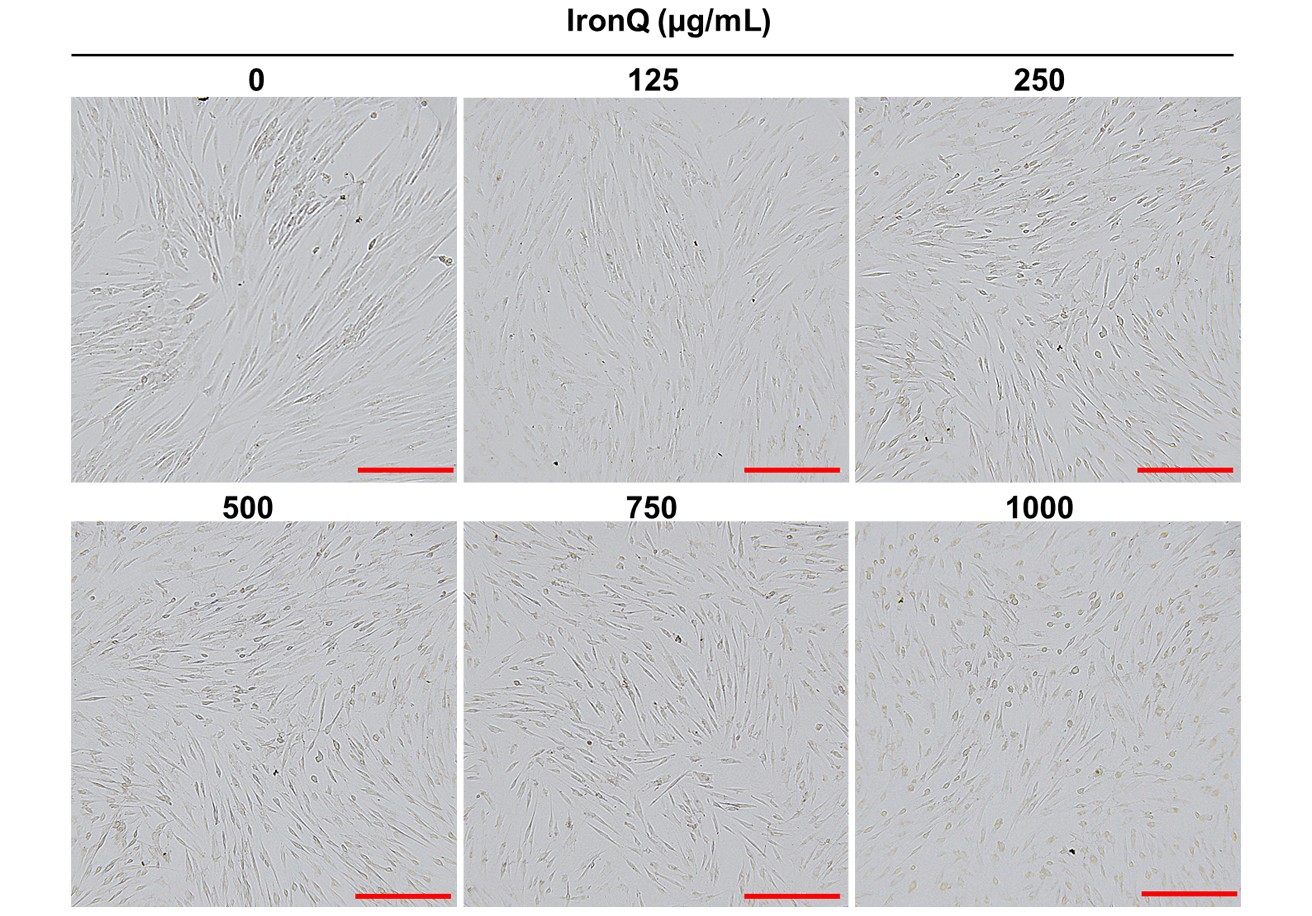


**Supplementary Figure S2**. Morphological effects of IronQ treatment on adipose-derived mesenchymal stem cells (ADSCs). Representative phase-contrast micrographs of ADSCs following 24 h exposure to increasing concentrations of IronQ (0–1000 µg mL⁻¹). ADSCs maintained the characteristic spindle-shaped fibroblast-like morphology of MSCs at biologically relevant non-cytotoxic concentrations (≤250 µg mL⁻¹), whereas higher concentrations were associated with reduced cell density and morphological alterations. Scale bar = 300 µm.


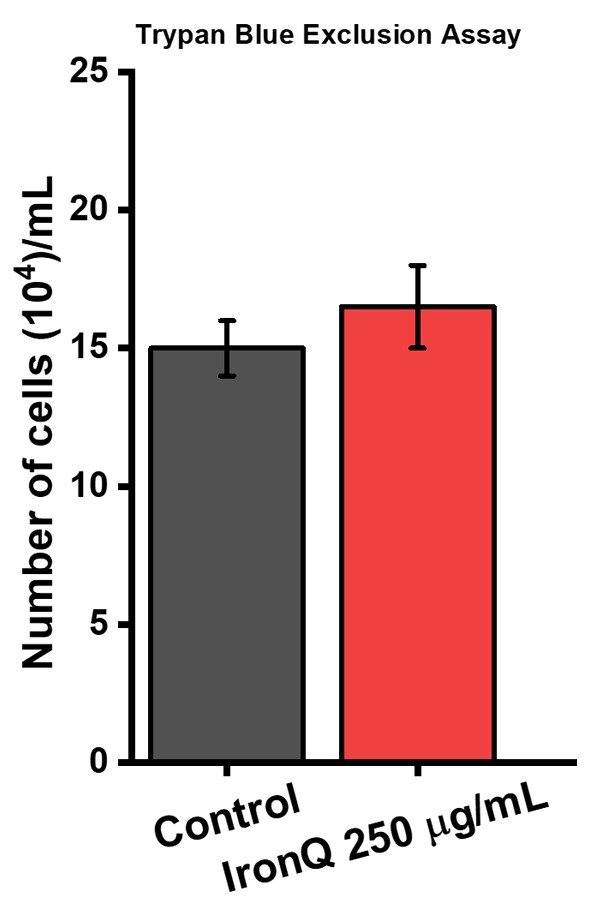


**Supplementary Figure S3**. Comparable ADSC cell number at the time of conditioned medium (CM) collection. Quantitative analysis of ADSC cell number determined by trypan blue exclusion assay in control and IronQ-treated (250 µg mL⁻¹) groups at the time of CM harvest. Data are presented as mean ± standard deviation (SD) from three independent experiments. No statistically significant differences in cell number were observed between groups (one-way ANOVA, *p* > 0.05).
